# Functional Rescue of CFTR-Dependent Transport in a Pancreatic Ductal Epithelial Cell Model: The Impact of Pharmacological Modulation and Inflammation

**DOI:** 10.3390/ijms27114868

**Published:** 2026-05-28

**Authors:** Alessandra Ludovico, Martina Battistini, Debora Baroni

**Affiliations:** Istituto di Biofisica, Consiglio Nazionale delle Ricerche (CNR), Via De Marini, 6, 16149 Genova, Italy; aleludo89@gmail.com (A.L.); martinabattistini1@gmail.com (M.B.)

**Keywords:** CFTR, CAPAN-1 cells, pancreatic duct epithelium, CFTR modulators, inflammation

## Abstract

Cystic fibrosis is a multi-organ disease in which pancreatic involvement occurs early and contributes significantly to disease progression. Despite this, most mechanistic and pharmacological studies of CFTR have been conducted in airway epithelia, while pancreatic duct models remain relatively poorly represented. In this study, we establish CAPAN-1 cells as a reproducible in vitro model of pancreatic duct epithelium and assess wild-type CFTR function under basal and inflammatory conditions. Cells were cultured as polarized monolayers and analysed for transepithelial conductance, ion transport, luminal fluid pH regulation, and microviscosity. CFTR activity was stimulated with forskolin and further modulated using the potentiator ivacaftor (VX770) and the correctors tezacaftor (VX661) and elexacaftor (VX445), while specificity was confirmed with the CFTR inhibitor PPQ102. Inflammation was induced by lipopolysaccharide (LPS). CAPAN-1 cells formed a functional epithelium. CFTR activation increased epithelial conductance, promoted apical surface fluid alkalinization, and reduced apical surface fluid microviscosity, while PPQ102 consistently inhibited these effects. CFTR modulators enhanced functional responses in the presence of forskolin, although with moderate magnitude, consistent with wild-type CFTR expression. LPS exposure altered epithelial properties, increasing baseline conductance and impairing pH regulation, and induced secretion of pro-inflammatory cytokines. Notably, inflammatory stimulation did not abolish CFTR modulator responses, although it modified some downstream epithelial outputs. These findings identify CAPAN-1 cells as a physiologically relevant model for investigating CFTR function in the pancreatic duct environment and show that CFTR modulator responses are maintained, although functionally reshaped, under inflammatory conditions.

## 1. Introduction

Cystic fibrosis (CF) is caused by mutations in the cystic fibrosis transmembrane conductance regulator (*CFTR*) gene, leading to defective epithelial chloride and bicarbonate transport [1,2]. Although pancreatic involvement represents a critical and early component of disease progression, it remains less extensively characterized than respiratory manifestations in in vitro studies [3,4,5,6]. CFTR plays a central role in pancreatic ductal epithelia by coordinating ion transport, fluid secretion, and luminal pH, processes that are essential for maintaining ductal clearance and preventing obstruction [7,8,9]. Alterations in these functions lead to profound changes in the physicochemical properties of the luminal environment [10]. Reduced bicarbonate secretion results in acidification, while impaired fluid transport promotes the accumulation of viscous secretions. These alterations lead to ductal obstruction, enzyme retention, and progressive tissue damage, ultimately resulting in exocrine pancreatic insufficiency, recurrent pancreatitis, and pancreatic fibrosis [11,12,13].

CFTR modulators are small molecules that improve CFTR protein expression, processing, trafficking, or channel activity, depending on the molecular defect targeted. Potentiators, such as ivacaftor (VX770), increase the open probability and functional activity of CFTR channels that are already present at the plasma membrane [14,15]. In contrast, correctors, such as tezacaftor (VX661) and elexacaftor (VX445), improve CFTR folding, maturation, and trafficking, thereby increasing the amount of functional protein at the cell surface, particularly in the case of folding-defective variants such as F508del-CFTR [16,17]. The combined use of potentiators and correctors has transformed CF therapy, with the triple combination elexacaftor/tezacaftor/ivacaftor approved as Kaftrio^®^ in Europe and Trikafta^®^ in the United States [18,19].

In addition to CFTR dysfunction, inflammation represents a key feature of CF-affected tissues and may further modulate epithelial physiology. Pro-inflammatory stimuli can alter barrier properties, interfere with epithelial transport, and reshape the luminal microenvironment. However, rather than acting solely as a detrimental factor, inflammation may differentially affect CFTR-dependent processes, potentially modifying the coordination between ion transport, fluid secretion, and luminal homeostasis. This aspect remains poorly defined in pancreatic epithelia, where the functional consequences of inflammation on CFTR-dependent outputs are still largely unexplored [20].

Most mechanistic studies on CFTR function have been performed in airway epithelial models, particularly human bronchial and nasal epithelial cells, which represent reference systems for studying CFTR-dependent transport and personalized responses to CFTR modulators in well-differentiated in vitro epithelia [21,22,23]. In contrast, mechanistic investigations of CFTR function in pancreatic ductal epithelia remain limited. Several pancreatic-derived epithelial cell models have been used to investigate CFTR-dependent or ductal ion transport mechanisms. CFPAC-1 cells, derived from a pancreatic adenocarcinoma of a patient with cystic fibrosis and carrying endogenous F508del-CFTR, represent a canonical CF pancreatic duct model for studying altered anion transport and pharmacological rescue of the CF phenotype [24,25,26]. Near-normal human pancreatic duct epithelial models, such as HPDE and HPDE6-E6E7 cells, preserve ductal features and have been used as non-malignant pancreatic duct epithelial systems [27]. Other pancreatic ductal adenocarcinoma-derived cell lines, including PANC-1, MIA PaCa-2, BxPC-3, and AsPC-1, are widely used to study pancreatic cancer biology, inflammatory signalling, and tumour-duct interactions, but their quantitative utility for CFTR-dependent epithelial transport is more limited [28]. In this framework, CAPAN-1 cells provide a relevant WT-CFTR pancreatic duct-derived model, since they retain epithelial and ductal features, form polarized monolayers on permeable supports, and support vectorial bicarbonate transport, making them suitable for integrated studies of pancreatic epithelial physiology [29].

In the present study, HBE cells were used as a reference model to benchmark CFTR-dependent epithelial function under basal and CFTR pharmacological stimulation conditions, while CAPAN-1 cells [30,31] were used to investigate WT-CFTR-dependent epithelial outputs in a pancreatic ductal context [32]. CAPAN-1 cells were exposed to a pro-inflammatory stimulus to model pancreatic ductal inflammation [33] and evaluate its impact on epithelial CFTR-mediated transport processes. Importantly, both CAPAN-1 and HBE cells expressing wild-type CFTR (WT-CFTR) were used to isolate the effects of pharmacological modulation and inflammation independently of mutation-specific defects. In this context, CFTR modulators were used as pharmacological tools to evaluate how CFTR activation and modulation affect integrated epithelial functions, rather than to correct a mutation-specific CFTR defect. This approach enables the identification of mechanisms that may contribute to epithelial dysfunction even in the presence of functional CFTR, as may occur in inflamed tissues.

To address these issues, we performed an integrated functional analysis combining complementary readouts [34]. Transepithelial conductance (ΔG) was used to assess ion transport, transepithelial fluid flux (J) to evaluate fluid reabsorption, and apical surface fluid pH to monitor bicarbonate secretion. These measurements were complemented by multiple particle tracking (MPT) analysis to quantify the microviscosity of the apical surface fluid. In addition, in CAPAN-1 epithelial models CFTR expression and inflammatory status were evaluated by Western blot analysis of CFTR and pro-inflammatory cytokines (IL-8, IL-6, and TNF-α) [35]. By analyzing these parameters under basal conditions and in the presence of LPS-induced inflammation, we aimed to determine whether pharmacological activation of CFTR restores a coordinated epithelial phenotype in pancreatic ductal epithelia and how inflammation modulates the relationship between CFTR activity and downstream functional outputs, including ion transport, fluid secretion, luminal fluid pH regulation, and microenvironmental properties.

## 2. Results

### 2.1. Epithelial Ion and Fluid Transport Is Enhanced by CFTR Activation and Partially Affected by Inflammation

Before pharmacological stimulation, HBE and CAPAN-1 epithelial layers displayed distinct baseline transepithelial conductance values. HBE monolayers showed a median G_pre_ of 1275.38 µS/cm^2^ (IQR: 1176.82–1491.29; *n* = 49), whereas CAPAN-1 monolayers showed higher basal conductance values, with a median G_pre_ of 6115.46 µS/cm^2^ (IQR: 5412.81–8544.28; *n* = 31).

**Figure 1:**
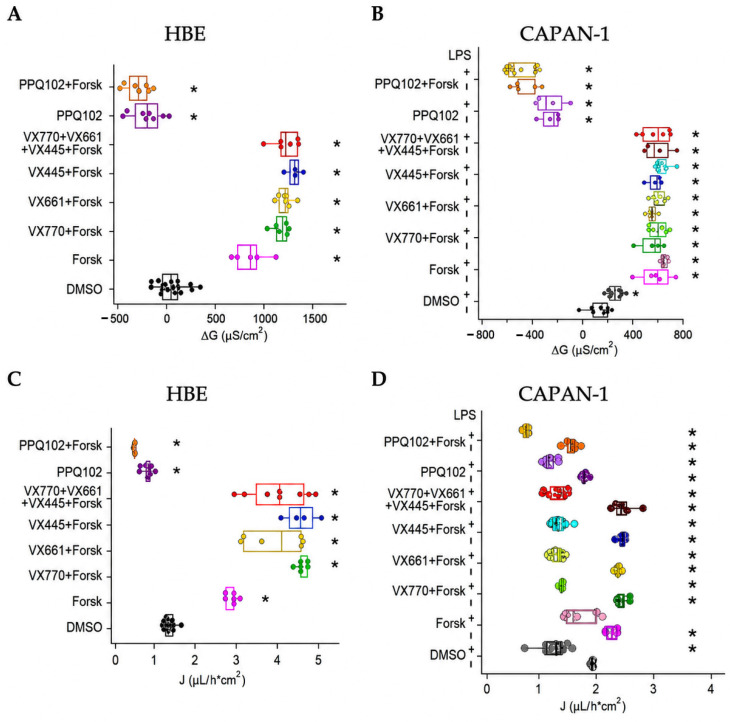
CFTR-dependent ion and fluid transport in HBE and CAPAN-1 epithelial models under basal conditions, pharmacological CFTR modulation, and pro-inflammatory stimulation. (**A**) Tran-sepithelial conductance (ΔG) in HBE cells under basal conditions (DMSO), after treatment with forskolin (Forsk), in the presence of CFTR modulators (VX770, VX661, VX445) combined with forskolin, or fol-lowing CFTR inhibition with PPQ102, alone or in combination with forskolin. (**B**) Transepithelial con-ductance (ΔG) in CAPAN-1 cells under basal conditions (DMSO), after treatment with forskolin, in the presence of CFTR modulators combined with forskolin, or following CFTR inhibition with PPQ102, alone or in combination with forskolin, in the absence (–) or presence (+) of LPS-induced pro-inflammatory stimulation. (**C**) Transepithelial fluid reabsorption (J) in HBE cells under basal and CFTR pharmaco-logical modulation, as described in (**A**). (**D**) Transepithelial fluid flux (J) in CAPAN-1 cells under basal, CFTR pharmacological modulation, and inflammation conditions as described in (**B**). Drug concentra-tions were as follows: forskolin (Forsk, 20 μM), VX770 (1 μM), VX661 (5 μM), VX445 (5 μM), PPQ102 (30 μM), and LPS (1 μg/mL), where applicable. Data are expressed as median with interquartile range (IQR). Each experimental condition included at least four independent epithelial samples (*n* ≥ 4). Statistical analysis was performed using the Kruskal–Wallis test followed by Dunn’s multiple comparison test. Comparisons were conducted versus the DMSO-treated, LPS-unstimulated control group. Asterisks indicate statistically significant differences (* *p* < 0.05) versus DMSO untreated samples.

In HBE epithelial layers (Figure 1A and Table S1), transepithelial conductance changes (ΔG, µS/cm^2^) markedly increased upon forskolin stimulation (median = 856.37, *n* = 5) compared with the basal DMSO-treated condition (median = 20.53, *n* = 12), suggesting robust CFTR activation. Forskolin in combination with CFTR modulators further enhanced ΔG, reaching median values of 1191.29 (*n* = 6), 1216.86 (*n* = 6), and 1322.10 (*n* = 4) for VX770, VX661, and VX445, respectively, while the triple combination yielded a median value of 1230.07 (*n* = 6). Inhibition with PPQ102 reduced ΔG to negative values both under basal (median = −204.53, *n* = 8) and stimulated conditions (median = −294.76, *n* = 7), confirming the specificity of CFTR-dependent ion transport.

In CAPAN-1 epithelial layers, DMSO-treated monolayers showed a median ΔG of 158.52, which increased upon LPS exposure (median = 288.21, *n* = 11), indicating partial modulation of epithelial transport under inflammatory conditions (Figure 1B and Table S2). Forskolin stimulation induced a marked increase in ΔG (median = 606.20, *n* = 5), which was preserved in the presence of LPS (median = 644.12, *n* = 6). CFTR modulators produced sustained high ΔG values under forskolin stimulation, with comparable levels observed under inflammatory conditions. In contrast, PPQ102 strongly reduced ΔG, yielding negative values both in basal (median = −221.36, *n* = 4) and stimulated conditions (median = −504.44, *n* = 5), with further reduction in the presence of LPS (median = −282.96, *n* = 4 and median = −535.33, *n* = 11, respectively).

A similar pattern was observed for apical fluid reabsorption (J, µL/(h·cm^2^)). In HBE epithelial layers (Figure 1C and Table S3), J increased from a median value of 1.38 (*n* = 12) under basal conditions to 2.94 (*n* = 6) following forskolin stimulation and reached values between 4.05 and 4.56 in the presence of CFTR modulators. In contrast, PPQ102 reduced J below basal levels (median = 0.91, *n* = 10) and further decreased it under stimulated conditions (median = 0.56, *n* = 7), indicating a tight coupling between CFTR activation and fluid transport in this model.

In CAPAN-1 epithelial layers (Figure 1D and Table S4), basal J had a median value of 2.07 (*n* = 10) and decreased in the presence of LPS (median = 1.35, *n* = 12). Forskolin increased J, whereas LPS reduced this response. CFTR modulators further increased J; however, all corresponding LPS conditions showed consistently lower values. PPQ102 reduced J under both basal and stimulated conditions (median = 1.90, *n* = 5 and 1.70, *n* = 5, respectively), with a further reduction in the presence of LPS (median = 1.16, *n* = 9 and 0.80, *n* = 5, respectively).

### 2.2. Apical Fluid pH Reflects CFTR-Dependent Bicarbonate Secretion

**Figure 2:**
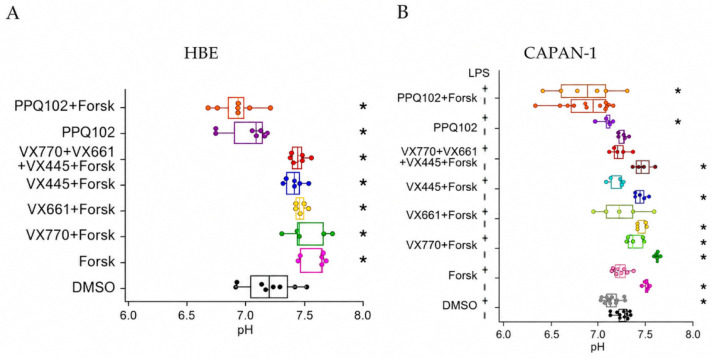
CFTR-dependent regulation of apical fluid pH in HBE and CAPAN-1 epithelial models under basal, pharmacological, and pro-inflammatory conditions. (**A**) Apical fluid pH in HBE epithelial cells under basal conditions (DMSO), following stimulation with forskolin (Forsk), in the presence of CFTR modulators (VX770, VX661, VX445) in combination with forskolin, or after CFTR inhibition with PPQ102 alone or combined with forskolin. (**B**) Apical fluid pH in CAPAN-1 epithelial cells under basal conditions (–) and following pro-inflammatory stimulation with LPS (+), in the presence or absence of CFTR activation (Forsk), CFTR modulators (VX770, VX661, VX445, and their triple combination), all in the presence of forskolin, or CFTR inhibition with PPQ102, alone or in combination with forskolin. Drug concentrations were as follows: forskolin (Forsk, 20 μM), VX770 (1 μM), VX661 (5 μM), VX445 (5 μM), PPQ102 (30 μM), and LPS (1 μg/mL), where applicable. Data are expressed as median with interquartile range (IQR). Each point represents an independent epithelial sample (*n* ≥ 4). Statistical analysis was performed using the Kruskal–Wallis test followed by Dunn’s multiple comparison test. Comparisons were conducted versus the DMSO-treated, LPS unstimulated condition within each experimental group. Asterisks indicate statistically significant differences (* *p* < 0.05).

In HBE epithelial cells, apical fluid pH under basal conditions was 7.24 (*n* = 9, Figure 2A). Forskolin stimulation significantly increased pH to 7.61 (*n* = 6), consistent with enhanced CFTR-dependent bicarbonate secretion. CFTR modulators, in combination with forskolin, did not further increase apical pH compared to forskolin alone, but maintained values within the same elevated range, with median values of 7.43, 7.45, and 7.42 for VX770, VX661, and VX445, respectively. The triple combination showed a comparable value (median = 7.48). In contrast, CFTR inhibition with PPQ102 significantly reduced pH under basal conditions (median = 7.07, *n* = 7) and further decreased it in the presence of forskolin (median = 6.94, *n* = 7, Figure 2A and Table S5).

In CAPAN-1 epithelial cells, basal apical pH was 7.30 (*n* = 12, Figure 2B). Forskolin stimulation significantly increased pH to 7.52 (*n* = 5). CFTR modulators, in combination with forskolin, maintained elevated pH values within a comparable range, including 7.62 for VX770 + Forsk, 7.43 for VX661 + Forsk, 7.45 for VX445 + Forsk, and 7.47 for the triple combination. CFTR inhibition with PPQ102 did not significantly affect basal pH (median = 7.28, *n* = 5), but reduced pH under forskolin-stimulated conditions (median = 7.00, *n* = 12).

Under pro-inflammatory conditions, LPS exposure significantly reduced apical pH compared to basal conditions (median = 7.17, *n* = 5). Across all treatments, pH values were consistently lower in the presence of LPS. In particular, the forskolin-induced increase in pH was attenuated (median = 7.26, *n* = 5). A similar reduction was observed for CFTR modulator conditions, including VX770 + Forsk + LPS (7.38), VX661 + Forsk + LPS (7.24), VX445 + Forsk + LPS (7.21), and the triple combination (7.22). CFTR inhibition further reduced pH under inflammatory conditions, with median values of 7.12 for PPQ102 + LPS and 6.94 for PPQ102 + Forsk + LPS (Figure 2B and Table S6).

### 2.3. CFTR Activation and Pro-Inflammatory Stimuli Regulate the Microviscosity of the Apical Fluid

Microviscosity measurements revealed a modulation of the apical fluid environment consistent with changes in CFTR activity in both HBE and CAPAN-1 epithelial layers.

**Figure 3:**
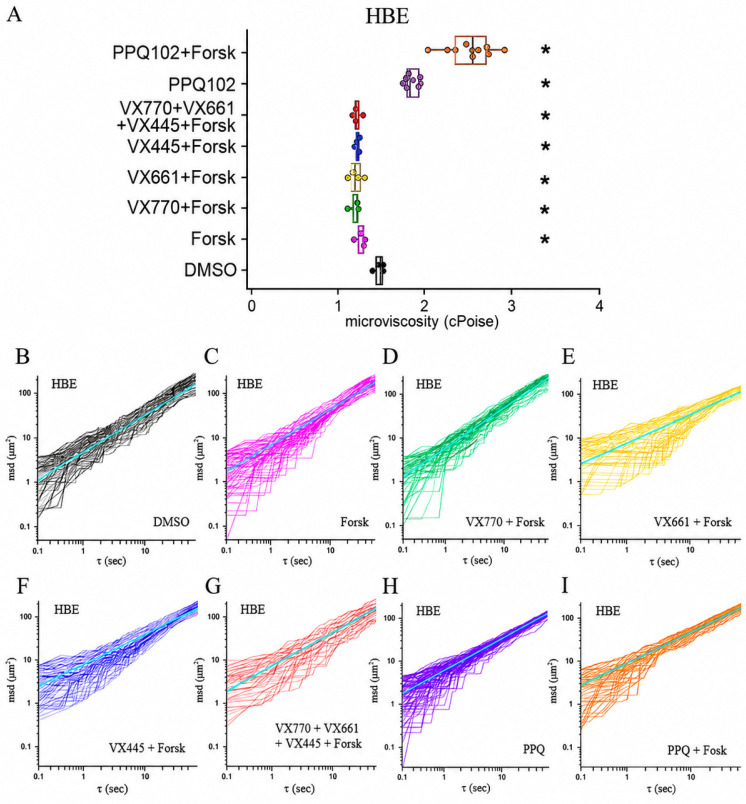
Microviscosity of the apical surface fluid of HBE epithelial layers as measured by multiple particle tracking (MPT). (**A**) Microviscosity of ASF collected from HBE layers treated with vehicle (0.1% DMSO), forskolin (Forsk, 20 μM), the CFTR potentiator VX770 (1 μM) in the presence of Forsk, the CFTR correctors VX661 (5 μM) and VX445 (5 μM) in combination with Forsk and the triple combination VX770 + VX661 + VX445 in the presence of forskolin, or the CFTR inhibitor PPQ102 (30 μM), in the absence or presence of Forsk. Data are expressed as median with interquartile range (IQR). Each point represents an independent epithelial sample (*n* ≥ 3). Statistical analysis was performed using the Kruskal–Wallis test followed by Dunn’s multiple comparison test. Comparisons were conducted versus the DMSO-treated condition. Asterisks indicate statistically significant differences (* *p* < 0.05). (**B**–**I**) Representative mean square displacement (MSD) curves plotted as a function of time interval (τ) for fluorescent tracer particles embedded in ASF under the experimental conditions indicated in each plot.

In HBE cells, the basal microviscosity (η, cPoise) value was 1.52 cPoise and decreased upon CFTR activation (Figure 3A, Table S7). Treatment with forskolin and VX770 in combination with forskolin reduced microviscosity to 1.31 and 1.25, respectively. A comparable reduction was also observed in the presence of CFTR correctors combined with forskolin, with VX661, VX445, and the triple combination (VX770 + VX661 + VX445) showing median values of 1.23, 1.26, and 1.24, respectively. In apical surface fluid samples in which CFTR was inhibited by PPQ102, microviscosity increased to 1.87, with a further increase observed in samples treated with PPQ102 in the presence of forskolin (2.56; Figure 3A–I, Table S7).

**Figure 4:**
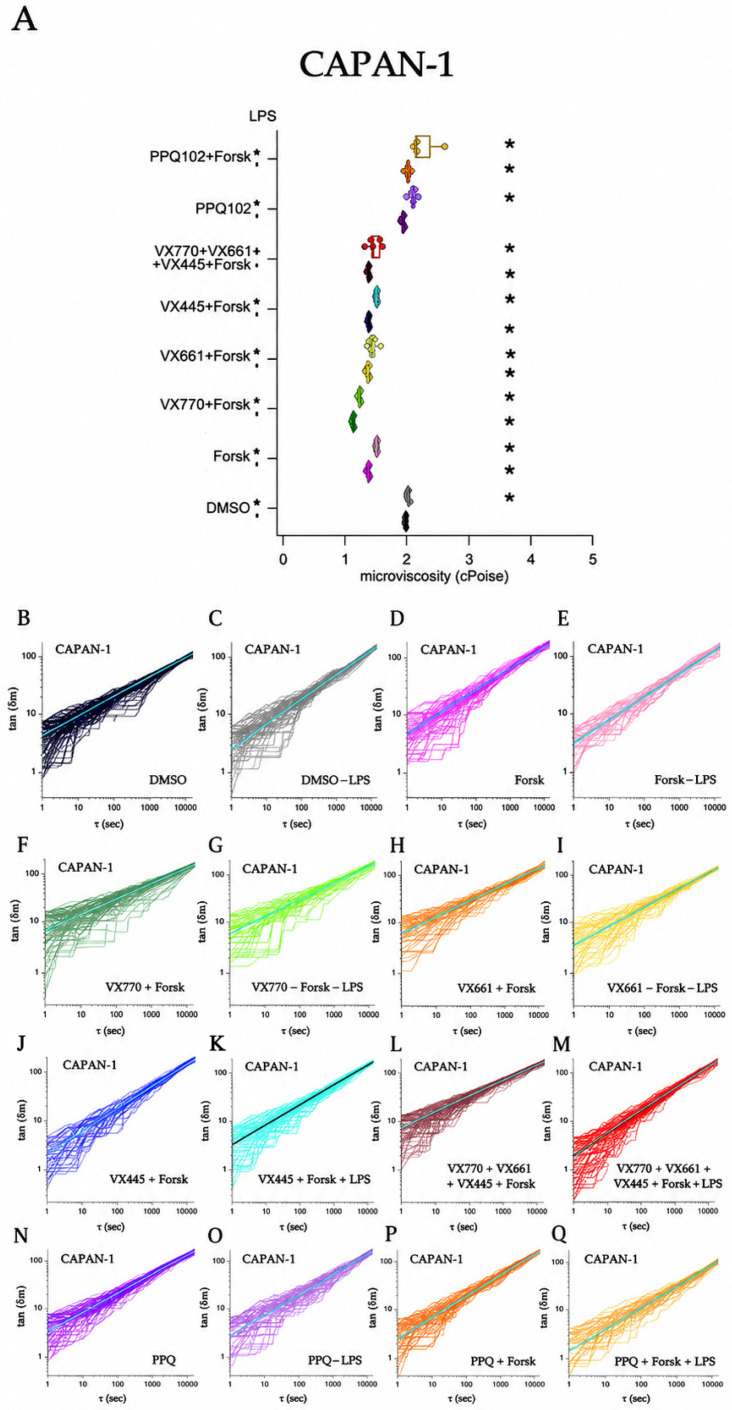
Microviscosity of apical surface fluid in CAPAN-1 epithelial layers as measured by multiple particle tracking (MPT). (**A**) Microviscosity of ASF collected from CAPAN-1 layers treated with vehicle (0.1% DMSO), forskolin (Forsk, 20 μM), the CFTR potentiator VX770 (1 μM) in the presence of Forsk, the CFTR correctors VX661 (5 μM) and VX445 (5 μM), alone or in combination, the triple combination VX770 + VX661 + VX445 in the presence of forskolin, or the CFTR inhibitor PPQ102 (30 μM), in the absence or presence of Forsk. Each condition was evaluated in the absence (–) or presence (+) of LPS. Data are expressed as median with interquartile range (IQR). Each point represents an independent epithelial sample (*n* ≥ 4). Statistical analysis was performed using the Kruskal–Wallis test followed by Dunn’s multiple comparison test. Comparisons were conducted versus the DMSO-treated condition. Asterisks indicate statistically significant differences (* *p* < 0.05). (**B**–**Q**) Representative mean square displacement (MSD) curves plotted as a function of time interval (τ) for fluorescent tracer particles embedded in ASF under the experimental conditions indicated in each plot.

Under basal conditions, median microviscosity values of the apical surface fluids of CAPAN-1 cells were 1.98 and increased following LPS exposure (2.03, *n* = 5). In apical fluid samples where CFTR was activated microviscosity decreased, with forskolin reducing median values to 1.39 and combined treatment with VX770 further reducing them to 1.13. In the presence of LPS, these effects were attenuated, with microviscosity values of 1.51 for forskolin and 1.25 for VX770 combined with forskolin (Figure 4A–G, Table S8).

Treatment with CFTR correctors in the presence of forskolin resulted in moderate changes in microviscosity. VX661 combined with forskolin showed values of 1.38, while VX445 combined with forskolin resulted in values comparable to forskolin alone (1.51, *n* = 5). The triple combination (VX770 + VX661 + VX445) in the presence of forskolin yielded intermediate values (1.39, *n* = 5). Under inflammatory conditions, these treatments were associated with slightly higher microviscosity values, including 1.48 for VX661 + forskolin and 1.39 for VX445 + forskolin, while the triple combination in the presence of LPS showed values of 1.46 (Figure 4A,H–M, Table S8).

CFTR inhibition by PPQ102 did not change microviscosity with respect to the DMSO-treated condition, whereas microviscosity increased in samples treated with PPQ102 in the presence of LPS, (2.10, *n* =5). Combined treatment with PPQ102 and forskolin yielded microviscosity median values of 2.03 and 2.17 in the absence and presence of LPS, respectively (Figure 4A,N–Q, Table S8).

Overall, microviscosity values of the apical surface fluid varied across conditions, with lower values observed following CFTR activation and higher values associated with CFTR inhibition and inflammatory stimulation (Figure 4A, Table S8).

### 2.4. CFTR Protein Expression Remains Largely Stable Across Pharmacological and Pro-Inflammatory Conditions

**Figure 5:**
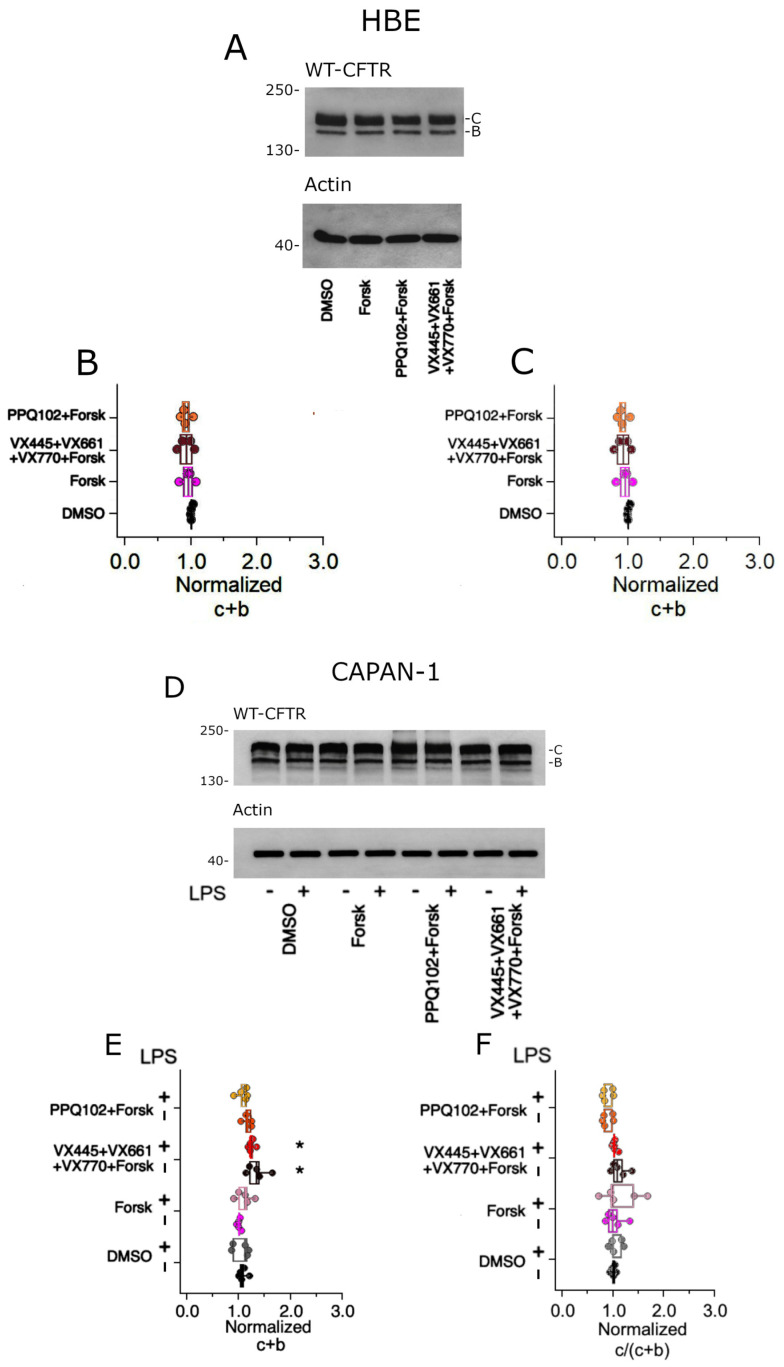
CFTR protein expression in HBE and CAPAN-1 epithelial models under basal, pharmacological, and inflammatory conditions. (**A**) Representative Western blot of WT-CFTR expression in HBE epithelial cells under basal conditions (DMSO), following stimulation with forskolin (Forsk, 20 μM), treatment with CFTR modulators (VX770 (1 μM) +VX661 (1 μM) +VX445 (1 μM)) in combination with forskolin, or CFTR inhibition with PPQ102 (30 μM) in the presence of forskolin. The lower panel shows actin as loading control. Molecular weight markers (kDa) are indicated on the left. The positions of the immature, core-glycosylated ER-resident form (band B) and the mature, fully glycosylated form (band C) of CFTR are indicated on the right. (**B**) Quantification of total CFTR expression (band C + band B) and (**C**) maturation rate (C/(C + B)) in HBE cells. (**D**) Representative Western blot of CFTR expression in CAPAN-1 epithelial cells under basal, pharmacological, and LPS-stimulated (1 μg/mL) conditions. The same concentrations of forskolin, CFTR modulators, and PPQ102 described in (**A**) were used. (**E**) Quantification of total CFTR expression (band C + band B) and (**F**) maturation rate (C/(C + B)) in CAPAN-1 cells. Band intensities were normalized to actin and expressed relative to DMSO-treated, LPS-unstimulated control samples, set to 1. Box plots represent median and interquartile range (IQR) from at least four independent experiments (*n* ≥ 4). Individual data points are shown as circles. Statistical analysis was performed using the Kruskal–Wallis test followed by Dunn’s multiple comparison test. Comparisons were conducted versus the DMSO-treated, LPS-unstimulated control group. Asterisks indicate statistically significant differences (* *p* < 0.05).

Western blot analysis showed that both HBE and CAPAN-1 epithelial preparations expressed detectable WT-CFTR protein, with the expected mature (band C) and immature (band B) forms present across all experimental conditions (Figure 5A,D). In HBE cells, CFTR expression remained stable following pharmacological treatments, with no evident treatment-dependent changes in the overall band pattern (Figure 5B,C and Table S9).

In CAPAN-1 cells (Figure 5D–F), basal CFTR expression was preserved across DMSO-, forskolin-, and PPQ102-treated conditions, indicating that neither CFTR activation nor inhibition significantly affected total protein abundance. Treatment with the triple combination VX770 + VX661 + VX445 in the presence of forskolin resulted in an increase in the mature form (band C), leading to a significant increase in total CFTR signal without a corresponding change in the maturation rate (C/(C + B)). A similar profile was observed under pro-inflammatory conditions (Figure 5E,F and Table S9).

Overall, these data indicate that CFTR expression remains largely stable across experimental conditions, supporting the conclusion that the functional effects observed are not associated with substantial changes in CFTR protein abundance.

### 2.5. LPS-Induced Pro-Inflammatory Cytokine Secretion Is Not Affected by CFTR Modulators in CAPAN-1 Epithelia

**Figure 6:**
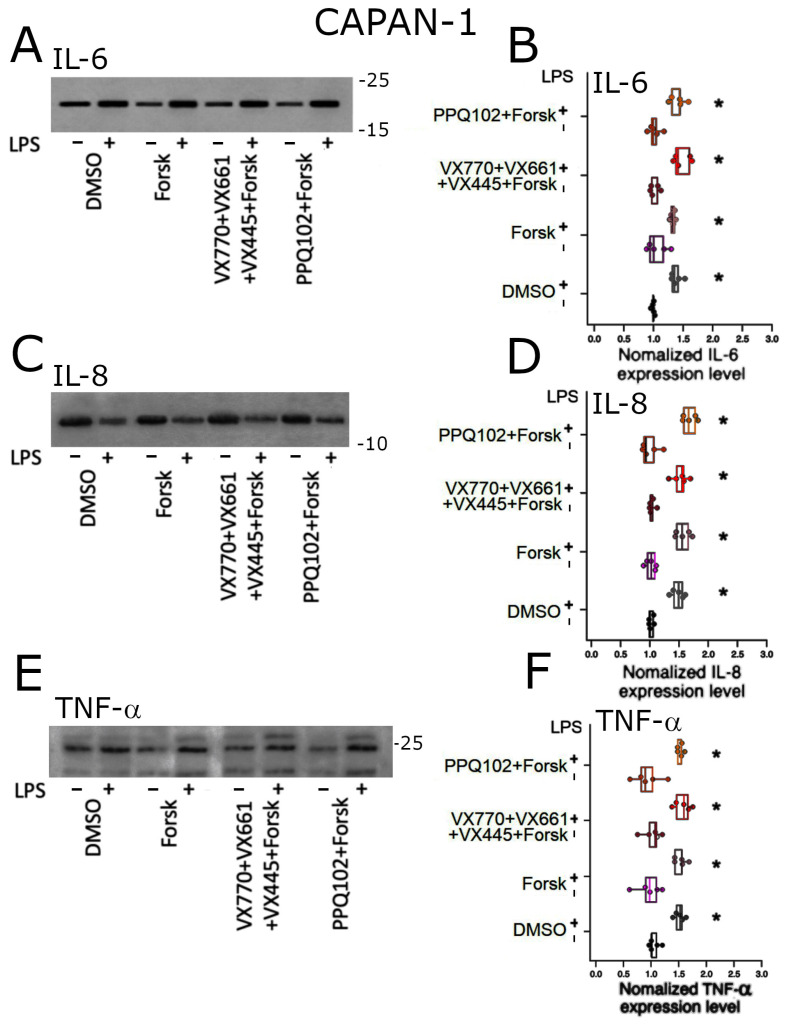
LPS-induced pro-inflammatory cytokine secretion is not affected by CFTR modulators in CAPAN-1 epithelial layers. (**A**,**C**,**E**) Representative Western blot analysis of IL-6 (**A**), IL-8 (**C**), and TNF-α (**E**) detected in concentrated basolateral media from CAPAN-1 epithelial cultures under basal conditions (–) and following LPS stimulation (+, 1 μg/mL), in the presence of forskolin (Forsk, 20 μM), CFTR modulators (VX770 (1 μM) +VX661 (5 μM) +VX445 (5 μM) + Forsk) or the inhibitor PPQ102 (30 μM) in combination with forskolin. (**B**,**D**,**F**) Semi-quantitative densitometric analysis of band intensities, normalized to Coomassie-stained gels (see Figure S2) and expressed relative to DMSO-treated, LPS-unstimulated control samples, set to 1. Data are presented as median with interquartile range (IQR). Each point represents an independent epithelial sample (*n* ≥ 4). Statistical analysis was performed using the Kruskal–Wallis test followed by Dunn’s multiple comparison test. Comparisons were conducted versus the DMSO-treated, LPS-unstimulated control group. Asterisks indicate statistically significant differences (* *p* < 0.05).

Under basal conditions, including CFTR stimulation with forskolin, treatment with the triple combination (VX770 + VX661 + VX445 + Forsk), or CFTR inhibition with PPQ102 + Forsk, the levels of IL-6, IL-8, and TNF-α detected in the concentrated basolateral medium of CAPAN-1 epithelial layers were low, indicating minimal basal inflammatory activity (Figure 6A–F and Table S10).

Following LPS stimulation, CAPAN-1 epithelial layers exhibited a marked increase in IL-6, IL-8, and TNF-α secretion into the basolateral medium compared to non-stimulated controls, confirming the activation of a robust pro-inflammatory response (Figure 6A–F). Cytokine levels remained elevated in all LPS-treated conditions, including those in the presence of CFTR modulators, and were comparable to those observed in DMSO-, forskolin-, and PPQ102-treated samples (Table S10).

These results indicate that, under the pro-inflammatory experimental conditions used (1 μg/mL LPS, 24 h), CFTR modulators do not exert detectable effects on IL-6, IL-8, and TNF-α production in CAPAN-1 epithelial layers.

## 3. Discussion

The study of CFTR function has been largely based on airway epithelial models, which have provided key insights into CFTR-dependent ion transport and pharmacological modulation [16,36,37]. However, cystic fibrosis is a multi-organ disease, and pancreatic involvement represents one of the earliest and clinically relevant manifestations [3,4,5]. Pancreatic damage is primarily driven by defective ductal secretion and altered luminal environments [38,39].

Despite this, pancreatic ductal epithelia remain underrepresented in mechanistic in vitro studies. In this context, identifying experimental models that retain key features of pancreatic epithelial physiology is essential. CAPAN-1 cells, although derived from a pancreatic adenocarcinoma, maintain epithelial characteristics and secretory features, including mucin expression, particularly MUC1 [30,31,40]. Here, we show that these cells form a polarized epithelium and support coordinated epithelial functions, including ion transport, fluid flux, pH regulation, and apical fluid layer organisation.

CAPAN-1 cells exhibited CFTR-dependent ion transport, as demonstrated by forskolin-induced increases in transepithelial conductance and their inhibition by PPQ102. Importantly, the limited effect of CFTR correctors in CAPAN-1 cells reflects the presence of WT-CFTR, which does not require rescue of folding or trafficking [41,42]. Under these conditions, CFTR function is primarily regulated at the level of channel activation, explaining the responsiveness to forskolin and VX770 and the minimal impact of corrector compounds. Although ΔG values were lower than in HBE cells, the overall response profile was consistent, indicating preserved CFTR function in a pancreatic context. These differences likely reflect tissue-specific specialization, as airway epithelia are optimized for chloride secretion, whereas pancreatic ducts rely on integrated chloride and bicarbonate transport mechanisms [43,44,45,46].

A central finding of this study is that CFTR-dependent processes are only partially coordinated in CAPAN-1 cells. In HBE epithelia, CFTR activation induced parallel increases in ion transport, fluid flux, and pH, consistent with a coordinated secretory response [46,47,48]. In contrast, CAPAN-1 cells showed a weaker coupling between these parameters, with fluid transport and pH changes less pronounced relative to ΔG [49,50]. This indicates that CFTR activation alone is not sufficient to fully drive epithelial secretion in pancreatic models, where additional regulatory pathways contribute to functional output.

Inflammation emerged as a key modulator of epithelial function [50,51,52,53,54]. In our CAPAN-1 epithelial preparations, LPS exposure increased basal conductance in CAPAN-1 monolayers, indicating altered epithelial transport properties [55,56]. Importantly, CFTR activation by forskolin was preserved under these conditions, suggesting that channel activity is not directly impaired. This observation is consistent with previous studies reporting maintained efficacy of CFTR activation in inflammatory environments [52,53,56,57,58,59].

In contrast, downstream functional outputs were markedly affected by inflammation. LPS reduced fluid flux, attenuated pH responses, and increased microviscosity, indicating that inflammation primarily disrupts epithelial secretion rather than CFTR channel activity. These findings support a model of functional uncoupling between CFTR-dependent ion transport and epithelial secretory processes under inflammatory conditions. Mechanistically, this may reflect alterations in epithelial barrier properties, osmotic gradients, and bicarbonate transport pathways [58,59,60].

Microviscosity measurements further support this interpretation. CFTR activation reduced microviscosity, consistent with improved apical surface fluid hydration, whereas inflammation impaired this effect, resulting in a less favourable luminal microenvironment despite preserved CFTR activity [61]. Western blot analysis supports a functional rather than quantitative interpretation. CFTR protein levels remained largely stable across treatments, with only a modest increase in mature CFTR after triple-combination treatment, indicating that the observed effects are mainly driven by modulation of channel activity rather than changes in protein abundance. Conversely, LPS induced a strong inflammatory response that was not attenuated by CFTR modulators, suggesting that epithelial dysfunction under inflammatory conditions cannot be fully corrected by targeting CFTR alone [53,58]. In this regard, it should be noted that Western blot analysis was used as a supportive readout to assess CFTR protein expression and maturation under different pharmacological and inflammatory conditions within each cell line preparation, rather than as a basis for direct quantitative comparison of CFTR abundance between HBE and CAPAN-1 cells. Indeed, establishing a rigorous inter-cell-line comparison would require additional approaches, including side-by-side protein analysis on the same membrane, and parallel CFTR mRNA quantification. These analyses were beyond the scope of the present study, which primarily relied on integrated functional CFTR-dependent readouts in in vitro CAPAN-1 pancreatic ductal epithelial preparations and on their sensitivity to CFTR-targeted pharmacological modulation and inflammatory stimulation.

Overall, these results confirm CFTR as a central regulator of epithelial physiology whose functional impact depends on the coordinated integration of multiple processes. This coordination is disrupted under inflammatory conditions, leading to a dissociation between channel activity and effective epithelial secretion [62].

## 4. Materials and Methods

### 4.1. Chemicals

Ivacaftor (VX770,N-(2,4-ditert-butyl-5-hydroxyphenyl)-4-oxo-1H-quinoline-3-carboxamide), tezacaftor (VX661,1-(2,2-difluoro-1,3-benzodioxol-5-yl)-N-[1-[(2R)-2,3-dihydroxypropyl]-6-fluoro2-(2-hydroxy-1,1-dimethylethyl)-1H-indol-5-yl]-cyclopropanecarboxamide), and elexacaftor (VX445,(S)-N-((1,3-dimethyl-1H-pyrazol-4-yl)sulfonyl)-6-(3-(3,3,3-trifluoro-2,2 dimethylpropoxy)1H-pyrazol-1-yl)-2-(2,2,4-trimethylpyrrolidin-1-yl)nicotinamide) were purchased from Selleck Chemicals (Munich, Germany). The CFTR inhibitor PPQ102 (12,14-dimethyl-9-(5methylfuran-2-yl)-17-phenyl 1,8,12,14 tetrazatetracyclo [8.7.0.02,7.011,16]heptadeca-2,4,6,10,16pentaene-13,15-dione) and forskolin (Forsk, 3R,4aR,5S,6S,6aS,10S,10aR,10bS-3-ethenyl-6,10,10btrihydroxy-3,4a,7,7,10a-pentamethyl-1-oxo-5,6,6a,8,9,10-hexahydro-2H-benzo[f]chromen-5-yl]acetate) were obtained from Merck (Milan, Italy). The chemical structures of VX770, VX661, VX445, and PPQ102 are reported in Figure S1. Lipopolysaccharide (LPS) from *Escherichia coli* O111:B4 was also purchased from Merck. All compounds were dissolved in dimethyl sulfoxide (DMSO) and diluted to the desired working concentrations in culture medium. The final DMSO concentration did not exceed 0.1% (*v*/*v*). Unless otherwise specified, all reagents were obtained from Merck.

### 4.2. Cell Culture

Human pancreatic ductal epithelial CAPAN-1 cells, originally isolated from the pancreas of a 40-year-old male with pancreatic adenocarcinoma [28,30,32], were commercially obtained from the American Type Culture Collection (ATCC, HTB-79, Manassas, VA, USA). Immortalized human bronchial epithelial cells (16HBE14o−, HBE [28,29,63]) were kindly provided by the Biobank of the IRCCS Istituto Giannina Gaslini, Genova, Italy. Both cell lines express wild-type (WT) CFTR. CAPAN-1 cells were cultured in high-glucose Dulbecco’s Modified Eagle’s Medium (DMEM) supplemented with 20% fetal bovine serum (FBS), whereas HBE were cultured in Minimum Essential Medium Eagle (MEM)/F12 (1:1) medium supplemented with 10% FBS. Both media contained 2 mM L-glutamine and 100 U/mL penicillin–streptomycin. Cells were maintained at 37 °C in a humidified atmosphere with 5% CO_2_.

Cells were seeded at high density (2.5 × 10^5^ cells per well) onto permeable polycarbonate Snapwell supports (0.4 µm pore size, 1.12 cm diameter; Corning, New York, NY, USA) pre-coated with rat tail collagen type I and cultured under liquid–liquid interface (LLI) conditions. The medium was replaced every 48 h until a stable and confluent epithelial layer was established (typically 10–15 days). Epithelial integrity was verified by measuring transepithelial electrical resistance (TEER) using an EVOM2 voltohmmeter (World Precision Instruments, Sarasota, FL, USA). For selected experiments, including fluid flux (J) and multiple particle tracking (MPT) analysis, cells were transitioned to air–liquid interface (ALI) conditions by removing the apical medium and maintaining cultures with the basolateral medium only for an additional 7–10 days, allowing apical surface exposure and fluid accumulation. Prior to experimental procedures, epithelial morphology, TEER stability, and overall monolayer integrity were verified.

### 4.3. Pharmacological Treatments and Inflammatory Stimulation

To assess CFTR-dependent epithelial function, cells were treated with CFTR modulators. Tezacaftor (VX661, 5 μM), elexacaftor (VX445, 5 μM), their combination, or the triple combination ivacaftor (VX770, 1 μM) + tezacaftor (VX661, 5 μM) + elexacaftor (VX445, 5 μM) were dissolved in 0.1% (*v*/*v*) DMSO and applied to the basolateral compartment for 24 h. To induce an inflammatory condition, lipopolysaccharide (LPS, 1 μg/mL) was added to the basolateral compartment 3 h prior to CFTR modulator treatment and maintained throughout the experiment. Ten minutes prior to functional measurements, cells (except DMSO controls) were acutely stimulated with forskolin (Forsk, 20 μM), applied to both apical and basolateral compartments to activate cAMP-dependent CFTR signaling. In selected conditions, the potentiator ivacaftor (VX770, 1 μM) was co-applied with forskolin to assess its potentiating effect, while CFTR inhibition was achieved by applying PPQ102 (30 μM) to both apical and basolateral compartments, either alone or 10 min after forskolin stimulation.

### 4.4. Measurement of Transepithelial Electrical Resistance (TEER) and Conductance (ΔG)

Transepithelial electrical resistance (TEER) was measured using an EVOM2 voltohmmeter (World Precision Instruments, Sarasota, FL, USA) equipped with STX2 chopstick electrodes [64]. Measurements were performed at 37 °C and normalized to the surface area of the insert (1.12 cm^2^), yielding TEER values expressed in Ω·cm^2^. Transepithelial conductance (G, µS/cm^2^) was calculated as the inverse of TEER (G = 1/TEER). Baseline conductance (G_pre_) was recorded immediately before stimulation, and post-stimulation conductance (G_post_) was measured 10 min after forskolin treatment. Changes in conductance are reported as ΔG = G_post_ − G_pre_. All experiments were performed in independent replicates (*n* ≥ 4 per condition), and data are presented as the median and interquartile range (IQR).

### 4.5. Measurement of Fluid Reabsorption (J)

To assess apical fluid reabsorption, 150 μL of isotonic buffer solution (NaCl 150 mM, HEPES 10 mM, pH 7.4) was applied to the apical surface of ALI-cultured epithelia. After 24 h of incubation at 37 °C and 5% CO_2_ in the presence of CFTR modulators, with or without LPS-induced pro-inflammatory stimulation, the residual apical fluid was collected and weighed in pre-weighed tubes. The final volume was calculated gravimetrically, assuming a density of 1 g/mL. The net fluid reabsorption rate (J) was calculated as follows:

J = (V_i_ − V_f_)/(At) (1)

where V_i_ = 150 μL, V_f_ is the final volume, A = 1.12 cm^2^, and t = 24 h.

Cell-free blank inserts were used to control for evaporation and pipetting variability. Results are expressed as μL·cm^−2^·h^−1^ and reported as the median and interquartile range (IQR, *n* ≥ 4 per condition). The method was adapted from previous studies employing gravimetric quantification of epithelial fluid transport [26].

### 4.6. pH Measurement in Apical Surface Fluid (ASF)

After 24 h of treatment with CFTR modulators, with or without LPS-induced pro-inflammatory stimulation, apical surface fluid (ASF) was collected from ALI-cultured epithelia, and its pH was measured using a microelectrode (SevenCompact, Mettler Toledo, Novate Milanese, Italy). To ensure pH stability, measurements were performed immediately after collection, and the time between sampling and analysis did not exceed 2 min. The instrument was calibrated using standard buffer solutions at pH 7.00 and pH 4.00. Each condition was tested in at least 4 independent replicates, and results are reported as the median and interquartile range (IQR). The method was adapted from previous studies employing gravimetric quantification of epithelial fluid transport [26,65,66].

### 4.7. Multiple Particle Tracking (MPT) Analysis

Multiple particle tracking (MPT) analysis was performed to evaluate the microrheological properties of ASF samples collected from ALI-cultured HBE and CAPAN-1 epithelial layers [65,66,67,68]. Briefly, 25 μL of ASF was mixed with 1 μL of yellow-green fluorescent carboxylated polystyrene beads (200 nm diameter; λ_ex_ = 488 nm; λ_em_ = 505–515 nm; Life Technologies, Monza, Italy). An aliquot of 8 μL of the mixture was sealed between glass coverslips to prevent evaporation and equilibrated for 20 min at room temperature. All measurements were performed at room temperature (22–24 °C), under identical conditions across samples. Beads were imaged at mid-height using a 60× oil-immersion objective (NA 1.42) coupled to a CCD camera. Videos (1280 × 960 pixels) were acquired at 5 frames/s.

Brownian trajectories were analysed using the Multitracker plugin in ImageJ software (version 1.53t, NIH), tracking approximately 400 particles from 4 to 8 fields per sample. The motion of the beads over a time interval τ was quantified by the mean squared displacement (MSD), from which the diffusion coefficient (D_0_) was derived according to:

⟨MSD(τ)⟩ = 4 D_0_ τα (2)

where τ is the time interval and α represents the elastic exponent of the medium (0 < α ≤ 1).

The microviscosity (η) was calculated using the Stokes–Einstein equation:

η = k_B_ T/(6π D_0_ r) (3)

where k_B_ is the Boltzmann constant, T is the absolute temperature, and r is the particle radius.

Microviscosity values were expressed in centipoise (cP), where 1 cP = 0.001 Pa·s, representing an estimate of the local microviscosity derived from particle diffusion.

### 4.8. Western Blot

Western blot analysis was performed on whole-cell lysates from HBE and CAPAN-1 epithelial cell preparations to evaluate CFTR protein expression and on concentrated basolateral media from CAPAN-1 epithelia to assess the secretion of pro-inflammatory cytokines IL-6, IL-8, and TNF-α. Briefly, for CFTR detection, CAPAN-1 and HBE epithelial layers were rinsed with PBS and detached by trypsinization. Cell pellets were then lysed in ice-cold RIPA buffer (50 mM Tris-HCl, pH 8.0, 150 mM NaCl, 1% Triton X-100, 1% sodium deoxycholate, 0.1% SDS) containing protease inhibitors. Lysates were mechanically homogenized by repeated passage through a syringe needle and subsequently sonicated using three cycles of 1 min each, with 3 min intervals on ice between cycles. Samples were centrifuged at 14,000× *g* at 4 °C for 15 min to remove cellular debris, and the supernatants were collected for protein analysis. Protein concentration was determined using the Bradford assay (Bio-Rad, Hercules, CA, USA), according to the manufacturer’s instructions. Aliquots containing 30 µg of total protein from whole-cell lysates were mixed with Laemmli sample buffer under reducing conditions and incubated at 37 °C for 30 min. Samples were not boiled before electrophoresis. Proteins were then resolved on 7% SDS-PAGE gels and transferred onto PVDF membranes (MilliporeSigma, Burlington, MA, USA). After transfer, membranes were incubated for 1 h at room temperature in blocking solution consisting of 5% bovine serum albumin (BSA) in PBS containing 0.1% Tween-20 (PBS-T). Membranes were then incubated overnight at 4 °C with anti-CFTR monoclonal antibody MM13–4 (1:200; MilliporeSigma), directed against the CFTR *n*-terminus and diluted in 5% BSA/PBS-T. After washing, membranes were re-blocked in 5% BSA in PBS-T for 1 h at room temperature and then incubated with HRP-conjugated goat anti-mouse secondary antibody (1:2000; Santa Cruz Biotechnology, Dallas, TX, USA) for 2 h at room temperature. Immunoreactive bands were detected using Amersham ECL Plus (GE Healthcare Europe GmbH, Milan, Italy) and visualized with Amersham Hyperfilm ECL (GE Healthcare/Cytiva, Marlborough, MA, USA). After CFTR detection, membranes were stripped and re-probed with anti-actin antibody (1:2000; Merck, Milan, Italy), used as loading control.

To detect secreted cytokines, basolateral media from CAPAN-1 epithelial cell preparations were collected and concentrated using Amicon Ultra-0.5 mL centrifugal filter units with a 3 kDa molecular weight cut-off at 14,000× *g* for 20 min at 4 °C. Protein concentration in both whole-cell lysates and concentrated basolateral samples was determined using the Bradford assay (Bio-Rad, Hercules, CA, USA), according to the manufacturer’s instructions. Aliquots corresponding to 30 μg of total protein from concentrated basolateral media were separated on 12% SDS-PAGE gels and transferred onto PVDF membranes (MilliporeSigma, Burlington, MA, USA). Membranes were blocked in 5% milk in PBS containing 0.1% Tween-20 (PBS-T) and incubated overnight at 4 °C with the following primary antibodies: CFTR (mouse monoclonal, 1:200, clone MM13–4; MilliporeSigma), IL-6 (rabbit polyclonal, 1:100; Thermo Fisher Scientific, Waltham, MA, USA), IL-8 (rabbit monoclonal, 1:100; Abcam, Cambridge, UK), or TNF-α (rabbit monoclonal, 1:100; Abcam). After washing, membranes were incubated with HRP-conjugated secondary antibodies (goat anti-mouse or anti-rabbit, 1:2000; Santa Cruz Biotechnology, Dallas, TX, USA) for 1 h at room temperature. Detection was performed using enhanced chemiluminescence ECL (Amersham ECL Plus, GE Healthcare), and signals were visualized using Hyperfilm ECL (GE Healthcare/Cytiva, Marlborough, MA, USA). To verify uniform protein loading and enable semi-quantitative normalization of secreted cytokines, total protein content in concentrated basolateral media was assessed by Coomassie Blue staining (R250, Bio-Rad) of SDS-PAGE gels. Band intensities were quantified using ImageJ software (version 1.53t, NIH, Bethesda, MD, USA). CFTR signals were normalized to actin from the same lane on the re-probed membranes, whereas cytokine signals were normalized to the corresponding Coomassie-stained lanes. For each target, results were expressed relative to the DMSO treated, LPS-unstimulated condition, which was set to 1. At least four independent biological replicates were analysed per condition.

### 4.9. Statistical Analysis

Data are presented as the median with the interquartile range (IQR). Each experiment was performed with 4–12 replicates per condition and independently repeated using at least three distinct cell culture passages to ensure biological reproducibility. All statistical and MPT analyses were performed using IgorPro 9 software (WaveMetrics, Lake Oswego, OR, USA). Differences between groups were assessed using the Kruskal–Wallis non-parametric ANOVA, followed by Dunn’s post hoc test for multiple comparisons. A value of *p* < 0.05 was considered statistically significant.

## 5. Conclusions

This study establishes CAPAN-1 cells as a functional epithelial model suitable for investigating WT-CFTR-dependent processes in a pancreatic context. CFTR activation induces robust ion transport that remains preserved under inflammatory conditions, indicating that channel activity is not directly impaired by LPS.

However, inflammation selectively disrupts downstream functional outputs, including fluid transport, pH regulation, and microviscosity, leading to a partial uncoupling between CFTR activity and epithelial secretion.

These findings highlight the importance of assessing CFTR function within an integrated epithelial framework and indicate that restoring CFTR activity alone may be insufficient to fully recover epithelial physiology in inflamed tissues.

## Data Availability

The original contributions presented in this study are included in the article/Supplementary Materials. Further inquiries can be directed to the corresponding author.

## Supplementary Materials

The following supporting information can be downloaded at: https://www.mdpi.com/article/10.3390/ijms27114868/s1.

## References

[1] Bobadilla J.L., Macek M., Fine J.P., Farrell P.M. (2002). Cystic fibrosis: A worldwide analysis of CFTR mutations, correlation with incidence data and application to screening. Hum. Mutat..

[2] Riordan J.R., Rommens J.M., Kerem B., Alon N., Rozmahel R., Grzelczak Z., Zielenski J., Lok S., Plavsic N., Chou J.L. (1989). Identification of the cystic fibrosis gene: Cloning and characterization of complementary DNA. Science.

[3] Gibson-Corley K.N., Meyerholz D.K., Engelhardt J.F. (2016). Pancreatic pathophysiology in cystic fibrosis. J. Pathol..

[4] Reid C.J., Hyde K., Ho S.B., Harris A. (1997). Cystic fibrosis of the pancreas: Involvement of MUC6 mucin in obstruction of pancreatic ducts. Mol. Med..

[5] Wilschanski M., Novak I. (2013). The cystic fibrosis of exocrine pancreas. Cold Spring Harb. Perspect. Med..

[6] Kim Y., Jun I., Shin D.H., Yoon J.G., Piao H., Jung J., Park H.W., Cheng M.H., Bahar I., Whitcomb D.C. (2020). Regulation of CFTR bicarbonate channel activity by WNK1: Implications for pancreatitis and CFTR-related disorders. Cell. Mol. Gastroenterol. Hepatol..

[7] Lukasiak A., Zajac M. (2021). The distribution and role of the CFTR protein in the intracellular compartments. Membranes.

[8] Angyal D., Bijvelds M.J.C., Bruno M.J., Peppelenbosch M.P., de Jonge H.R. (2021). Bicarbonate transport in cystic fibrosis and pancreatitis. Cells.

[9] Ishiguro H., Steward M.C., Naruse S., Ko S.B., Goto H., Case R.M., Kondo T., Yamamoto A. (2009). CFTR functions as a bicarbonate channel in pancreatic duct cells. J. Gen. Physiol..

[10] McCague A.F., Raraigh K.S., Pellicore M.J., Davis-Marcisak E.F., Evans T.A., Han S.T., Lu Z., Joynt A.T., Sharma N., Castellani C. (2019). Correlating cystic fibrosis transmembrane conductance regulator function with clinical features to inform precision treatment of cystic fibrosis. Am. J. Respir. Crit. Care Med..

[11] Durie P.R., Forstner G.G. (1989). Pathophysiology of the exocrine pancreas in cystic fibrosis. J. R. Soc. Med..

[12] Karpińska M., Czauderna M. (2022). Pancreas: Its functions, disorders, and physiological impact on the mammals’ organism. Front. Physiol..

[13] Hegyi P., Seidler U., Kunzelmann K. (2023). CFTR-beyond the airways: Recent findings on the role of the CFTR channel in the pancreas, the intestine and the kidneys. J. Cyst. Fibros..

[14] Van Goor F., Hadida S., Grootenhuis P.D., Burton B., Cao D., Neuberger T., Turnbull A., Singh A., Joubran J., Hazlewood A. (2009). Rescue of CF airway epithelial cell function in vitro by a CFTR potentiator, VX-770. Proc. Natl. Acad. Sci. USA.

[15] Eckford P.D.W., Li C., Ramjeesingh M., Bear C.E. (2012). Cystic fibrosis transmembrane conductance regulator (CFTR) potentiator VX-770 (ivacaftor) opens the defective channel gate of mutant CFTR in a phosphorylation-dependent but ATP-independent manner. J. Biol. Chem..

[16] Donaldson S.H., Pilewski J.M., Griese M., Cooke J., Viswanathan L., Tullis E., Davies J.C., Lekstrom-Himes J.A., Wang L.T. (2018). Tezacaftor/Ivacaftor in Subjects with Cystic Fibrosis and F508del/F508del-CFTR or F508del/G551D-CFTR. Am. J. Respir. Crit. Care Med..

[17] Veit G., Roldan A., Hancock M.A., Da Fonte D.F., Xu H., Hussein M., Frenkiel S., Matouk E., Velkov T., Lukacs G.L. (2020). Allosteric folding correction of F508del and rare CFTR mutants by elexacaftor-tezacaftor-ivacaftor (Trikafta) combination. JCI Insight.

[18] European Medicines Agency *Kaftrio*: EPAR, Product Information. https://www.ema.europa.eu/en/medicines/human/EPAR/kaftrio.

[19] U.S. Food and Drug Administration *TRIKAFTA^®^* (Elexacaftor, Tezacaftor, and Ivacaftor Tablets; Ivacaftor Tablets), Co-Packaged for Oral Use: Prescribing Information. https://www.google.com/url?sa=t&source=web&rct=j&opi=89978449&url=https://www.accessdata.fda.gov/drugsatfda_docs/label/2021/212273s004lbl.pdf&ved=2ahUKEwjy9uffgduUAxUVxDgGHQivAi4QFnoECB8QAQ&usg=AOvVaw2UYYYAj26F4HIQSj0wGeJi.

[20] Madácsy T., Pallagi P., Maleth J. (2018). Cystic fibrosis of the pancreas: The role of CFTR channel in the regulation of intracellular Ca^2+^ signaling and mitochondrial function in the exocrine pancreas. Front. Physiol..

[21] Pion A., Kavanagh E., Joynt A.T., Raraigh K.S., Vanscoy L., Langfelder-Schwind E., McNamara J., Moore B., Patel S., Merlo K. (2024). Investigation of CFTR Function in Human Nasal Epithelial Cells Informs Personalized Medicine. Am. J. Respir. Cell Mol. Biol..

[22] Awatade N.T., Wong S.L., Hewson C.K., Fawcett L.K., Kicic A., Jaffe A., Waters S.A. (2018). Human primary epithelial cell models: Promising tools in the era of cystic fibrosis personalized medicine. Front. Pharmacol..

[23] Pranke I., Capurro V., Chevalier B., Pesce E., Tomati V., Pastorino C., Kelly-Aubert M., Hatton A., Dreano E., Lena M. (2025). Beyond Trikafta: New models to assess tissue dependent rescue of N1303K-CFTR. Front. Pharmacol..

[24] Ludovico A., Battistini M., Baroni D. (2026). Cystic Fibrosis of the Pancreas: In Vitro Duct Models for CFTR-Targeted Translational Research. Int. J. Mol. Sci..

[25] Schoumacher R.A., Ram J., Iannuzzi M.C., Bradbury N.A., Wallace R.W., Hon C.T., Kelly D.R., Schmid S.M., Gelder F.B., Rado T.A. (1990). A cystic fibrosis pancreatic adenocarcinoma cell line. Proc. Natl. Acad. Sci. USA.

[26] Ludovico A., Baroni D. (2025). CFTR Modulators Counteract F508del CFTR Functional Defects in a Pancreatic Epithelial Model of Cystic Fibrosis. Life.

[27] Ouyang H., Mou L.J., Luk C., Liu N., Karaskova J., Squire J., Tsao M.S. (2000). Immortal human pancreatic duct epithelial cell lines with near normal genotype and phenotype. Am. J. Pathol..

[28] Deer E.L., González-Hernández J., Coursen J.D., Shea J.E., Ngatia J., Scaife C.L., Firpo M.A., Mulvihill S.J. (2010). Phenotype and genotype of pancreatic cancer cell lines. Pancreas.

[29] Ishiguro H., Naruse S., San Román J.I., Case M., Steward M.C. (2001). Pancreatic ductal bicarbonate secretion: Past, present and future. JOP.

[30] Kyriazis A.P., Kyriazis A.A., Scarpelli D.G., Fogh J., Rao M.S., Lepera R. (1982). Human pancreatic adenocarcinoma line Capan-1 in tissue culture and the nude mouse: Morphologic, biologic, and biochemical characteristics. Am. J. Pathol..

[31] Szucs A., Demeter I., Burghardt B., Ovári G., Case R.M., Steward M.C., Varga G. (2006). Vectorial bicarbonate transport by Capan-1 cells: A model for human pancreatic ductal secretion. Cell. Physiol. Biochem..

[32] Hollande E., Fanjul M., Chemin-Thomas C., Devaux C., Demolombe S., Van Rietschoten J., Guy-Crotte O., Figarella C. (1998). Targeting of CFTR protein is linked to the polarization of human pancreatic duct cells in culture. Eur. J. Cell Biol..

[33] Blanchard J.A., Barve S., Joshi-Barve S., Talwalker R., Gates L.K. (2000). Cytokine production by CAPAN-1 and CAPAN-2 cell lines. Dig. Dis. Sci..

[34] Doryab A., Schmid O. (2022). Towards a gold standard functional readout to characterize in vitro lung barriers. Eur. J. Pharm. Sci..

[35] Roshani R., McCarthy F., Hagemann T. (2014). Inflammatory cytokines in human pancreatic cancer. Cancer Lett..

[36] Fulcher M.L., Randell S.H. (2013). Human nasal and tracheo-bronchial respiratory epithelial cell culture. Methods Mol. Biol..

[37] Callaghan P.J., Ferrick B., Rybakovsky E., Thomas S., Mullin J.M. (2020). Epithelial barrier function properties of the 16HBE14o- human bronchial epithelial cell culture model. Biosci. Rep..

[38] Freedman S.D., Blanco P.G., Zaman M.M., Shea J.C., Ollero M., Hopper I.K., Weed D.A., Gelrud A., Regan M.M., Laposata M. (2004). Association of cystic fibrosis with abnormalities in fatty acid metabolism. N. Engl. J. Med..

[39] Rowe S.M., Miller S., Sorscher E.J. (2005). Cystic fibrosis. N. Engl. J. Med..

[40] Qu C.F., Li Y., Song Y.J., Rizvi S.M., Raja C., Zhang D., Samra J., Smith R., Perkins A.C., Apostolidis C. (2004). MUC1 expression in primary and metastatic pancreatic cancer cells for in vitro treatment by (213)Bi-C595 radioimmunoconjugate. Br. J. Cancer.

[41] Gentzsch M., Chang X.B., Cui L., Wu Y., Ozols V.V., Choudhury A., Pagano R.E., Riordan J.R. (2004). Endocytic trafficking routes of wild type and DeltaF508 cystic fibrosis transmembrane conductance regulator. Mol. Biol. Cell.

[42] Amico G., Brandas C., Moran O., Baroni D. (2019). Unravelling the regions of mutant F508del CFTR more susceptible to the action of four cystic fibrosis correctors. Int. J. Mol. Sci..

[43] Kim D., Steward M.C. (2009). The role of CFTR in bicarbonate secretion by pancreatic duct and airway epithelia. J. Med. Investig..

[44] Novak I., Hansen M.R. (2002). Where have all the Na+ channels gone? In search of functional ENaC in exocrine pancreas. Biochim. Biophys. Acta.

[45] Boucher R.C. (2007). Cystic fibrosis: A disease of vulnerability to airway surface dehydration. Trends Mol. Med..

[46] Saint-Criq V., Gray M.A. (2017). Role of CFTR in epithelial physiology. Cell. Mol. Life Sci..

[47] Rakonczay Z., Fearn A., Hegyi P., Boros I., Gray M.A., Argent B.E. (2006). Characterization of H^+^ and HCO^3-^ transporters in CFPAC-1 human pancreatic duct cells. World J. Gastroenterol..

[48] Sørensen C.E., Trauzold A., Christensen N.M., Tawfik D., Szczepanowski M., Novak I. (2023). Synergistic effects of agonists and two-pore-domain potassium channels on secretory responses of human pancreatic duct cells Capan-1. Pflug. Arch..

[49] Park H.W., Lee M.G. (2012). Transepithelial bicarbonate secretion: Lessons from the pancreas. Cold Spring Harb. Perspect. Med..

[50] Pooran N., Indaram A., Singh P., Bank S. (2003). Cytokines (IL-6, IL-8, TNF): Early and reliable predictors of severe acute pancreatitis. J. Clin. Gastroenterol..

[51] Angyal D., Groeneweg T.A., Leung A., Desain M., Dulla K., de Jonge H.R., Bijvelds M.J.C. (2024). Pro-inflammatory cytokines stimulate CFTR-dependent anion secretion in pancreatic ductal epithelium. Cell. Mol. Biol. Lett..

[52] Hisert K.B., Heltshe S.L., Pope C., Jorth P., Wu X., Edwards R.M., Radey M., Accurso F.J., Wolter D.J., Cooke G. (2017). Restoring cystic fibrosis transmembrane conductance regulator function reduces airway bacteria and inflammation in people with cystic fibrosis and chronic lung infections. Am. J. Respir. Crit. Care Med..

[53] Ribeiro C.M.P., Gentzsch M. (2021). Impact of airway inflammation on the efficacy of CFTR modulators. Cells.

[54] Wu Y., Konate M., Hollingshead M., Karim B., Diebold B., Lu J., Antony S., Meitzler J.L., Juhasz A., Jiang G. (2022). Dexamethasone inhibits cytokine-induced, DUOX2-related VEGF-A expression and DNA damage in human pancreatic cancer cells and growth of pancreatic cancer xenografts. bioRxiv.

[55] Sivam H.G.P., Chin B.Y., Gan S.Y., Ng J.H., Gwenhure A., Chan E.W.L. (2023). Lipopolysaccharide stimulation of pancreatic ductal adenocarcinoma and macrophages activates the NLRP3 inflammasome that influences the levels of pro-inflammatory cytokines in a co-culture model. Cancer Biol. Ther..

[56] Zhang X., Tian X., Wang Y., Yan Y., Wang Y., Su M., Lv H., Li K., Hao X., Xing X. (2024). Application of lipopolysaccharide in establishing inflammatory models. Int. J. Biol. Macromol..

[57] Bruscia E.M., Bonfield T.L. (2016). Innate and adaptive immunity in cystic fibrosis. Clin. Chest Med..

[58] Rehman T., Pezzulo A.A., Thurman A.L., Zemans R.L., Welsh M.J. (2024). Epithelial responses to CFTR modulators are improved by inflammatory cytokines and impaired by antiinflammatory drugs. JCI Insight.

[59] Jarosz-Griffiths H.H., Scambler T., Wong C.H., Lara-Reyna S., Holbrook J., Martinon F., Savic S., Whitaker P., Etherington C., Spoletini G. (2020). Different CFTR modulator combinations downregulate inflammation differently in cystic fibrosis. eLife.

[60] Rotoli B.M., Bussolati O., Dall’Asta V., Orlandini G., Gatti R., Gazzola G.C. (2000). Secretin increases the paracellular permeability of CAPAN-1 pancreatic duct cells. Cell. Physiol. Biochem..

[61] Constantinescu A.A., Gleizes C., Alhosin M., Yala E., Zobairi F., Leclercq A., Stoian G., Mitrea I.L., Prévost G., Toti F. (2014). Exocrine cell-derived microparticles in response to lipopolysaccharide promote endocrine dysfunction in cystic fibrosis. J. Cyst. Fibros..

[62] Chanson M., Scerri I., Suter S. (1999). Defective regulation of gap junctional coupling in cystic fibrosis pancreatic duct cells. J. Clin. Investig..

[63] Forbes B., Shah A., Martin G.P., Lansley A.B. (2003). The human bronchial epithelial cell line 16HBE14o- as a model system of the airways for studying drug transport. Int. J. Pharm..

[64] Srinivasan B., Kolli A.R., Esch M.B., Abaci H.E., Shuler M.L., Hickman J.J. (2015). TEER measurement techniques for in vitro barrier model systems. J. Lab. Autom..

[65] Gianotti A., Capurro V., Delpiano L., Mielczarek M., García-Valverde M., Carreira-Barral I., Ludovico A., Fiore M., Baroni D., Moran O. (2020). Small molecule anion carriers correct abnormal airway surface liquid properties in cystic fibrosis airway epithelia. Int. J. Mol. Sci..

[66] Ludovico A., Moran O., Baroni D. (2022). Modulator combination improves in vitro the microrheological properties of the airway surface liquid of cystic fibrosis airway epithelia. Int. J. Mol. Sci..

[67] Qian H., Sheetz M.P., Elson E.L. (1991). Single particle tracking. Analysis of diffusion and flow in two-dimensional systems. Biophys. J..

[68] Wirtz D. (2009). Particle-tracking microrheology of living cells: Principles and applications. Annu. Rev. Biophys..

