# Supplementary material for: Functional Rescue of CFTR-Dependent Transport in a Pancreatic Ductal Epithelial Cell Model: The Impact of Pharmacological Modulation and Inflammation
Source: Int J Mol Sci. 2026 May 28;27(11):4868. doi: 10.3390/ijms27114868 (PMC13256138; doi:10.3390/ijms27114868)

Functional Rescue of CFTR-Dependent Transport in a pancreatic ductal epithelial cell model: Impact of Pharmacological Modulation and Inflammation

Supplementary Materials

Alessandra Ludovico, Martina Battistini and Debora Baroni *
Istituto di Biofisica, National Research Council (CNR), Via De Marini, 6, 16149 Genova, Italy;

* Correspondence: debora.baroni@ibf.cnr.it; Tel.: +39-0106475559; Fax: +39-0106475500

Table S1. Transepithelial conductance (ΔG) in HBE epithelial models under basal and pharmacological modulation conditions. Data are reported as median, interquartile range (IQR25-IQR75), minimum (MIN), and maximum (MAX) values. HBE cells were treated with vehicle (DMSO), forskolin (Forsk), CFTR modulators (VX770, VX661, VX445) in combination with forskolin, or the CFTR inhibitor PPQ102, alone or in combination with forskolin. Sample size (n) and p values (Kruskal-Wallis test followed by Dunn's multiple comparison test) are indicated. Comparisons were performed using DMSO conditions as reference. Statistical significance was set at $p < 0.05$.

**ΔG in HBE epithelial models (µS/cm²)**							
**TREATMENT**	**MEDIAN**	**IQR25**	**IQR75**	**MIN**	**MAX**	**n**	**p**
DMSO	20.53	-57.83	121.35	-168.33	337.91	16	-
Forsk	856.37	717.48	924.04	658.92	1122.32	5	0.00045
VX770 + Forsk	1191.29	1127.71	1236.90	1021.22	1258.86	6	0.00083
VX661 + Forsk	1216.86	1161.77	1245.94	1102.68	1348.50	6	0.00083
VX445 + Forsk	1322.10	1294.20	1344.19	1210.50	1410.46	4	0.00151
VX445 + VX661 + VX770 + Forsk	1230.07	1176.79	1340.31	992.55	1367.08	6	0.00083
PPQ102	-204.53	-294.70	-118.70	-456.18	18.18	8	0.00182
PPQ102 + Forsk	-294.76	-392.16	-207.02	-493.40	-146.66	7	0.00051

Table S2. Transepithelial conductance (ΔG) in CAPAN-1 epithelial models under basal, inflammatory, and pharmacological modulation conditions. Data are reported as median, interquartile range (IQR25-IQR75), minimum (MIN), and maximum (MAX) values. CAPAN-1 cells were treated with vehicle (DMSO), lipopolysaccharide (LPS), forskolin (Forsk), CFTR modulators (VX770, VX661, VX445) in combination with forskolin, or the CFTR inhibitor PPQ102, in the presence or absence of Forsk and LPS. Sample size (n) and p values (Kruskal-Wallis test followed by Dunn's multiple comparison test) are indicated. Comparisons were performed using DMSO conditions as reference. Statistical significance was set at $p < 0.05$.

ΔG in CAPAN-1 epithelial models (µS/cm²)							
**TREATMENT**	**Median**	**IQR25**	**IQR75**	**MIN**	**MAX**	**n**	**p**
DMSO	158.52	82.76	198.78	-29.18	236.32	8	-
DMSO + LPS	288.21	222.09	290.46	170.17	353.31	11	0.00344
Forsk	606.20	543.97	684.94	590.96	690.14	5	0.00441
Forsk + LPS	644.12	631.04	673.70	618.22	680.67	6	0.00050
VX770 + Forsk	576.25	517.03	624.06	553.10	631.10	4	0.00441
VX770 + Forsk + LPS	602.57	544.10	632.80	534.69	632.80	6	0.00050
VX661 + Forsk	553.90	541.34	581.67	500.27	625.27	4	0.00441
VX661 + Forsk + LPS	606.13	575.56	655.44	531.17	655.44	6	0.00045
VX445 + Forsk	596.77	558.50	621.60	579.53	635.35	4	0.00441
VX445 + Forsk + LPS	617.28	595.74	666.37	594.42	666.37	6	0.00050
VX770 + VX661 + VX445 + Forsk	573.53	519.96	640.76	527.38	651.55	4	0.00441
VX770 + VX661 + VX445 + Forsk + LPS	606.50	504.91	646.62	434.85	655.37	6	0.00050
PPQ102	-221.36	-281.19	-185.31	-299.11	-157.41	4	0.00441
PPQ102+LPS	-282.96	-343.77	-157.24	-365.77	-145.33	4	0.0050
PPQ102+Forsk	-504.44	-513.03	-457.37	-572.34	-513.03	5	0.00141
PPQ102+Forsk+LPS	-535.33	-572.25	-362.14	-609.01	-335.27	11	0.00019

Table S3. Transepithelial fluid flux (J) in HBE epithelial models under basal and pharmacological modulation conditions. Data are reported as median, interquartile range (IQR25–IQR75), minimum (MIN), and maximum (MAX) values. HBE cells were treated with vehicle (DMSO), forskolin (Forsk), CFTR modulators (VX770, VX661, VX445) in combination with forskolin, or the CFTR inhibitor PPQ102 in the presence or absence of Forsk. Sample size (n) and p values (Kruskal–Wallis test followed by Dunn's multiple comparison test) are indicated. Comparisons were performed against the corresponding DMSO condition within each experimental group. Statistical significance was set at $p < 0.05$.

Transepithelial fluid flux (J) in HBE epithelial models (µL/(h·cm²))							
**TREATMENT**	**Median**	**IQR25**	**IQR75**	**MIN**	**MAX**	**n**	**p**
DMSO	1.38	1.31	1.48	1.23	1.69	12	-
Forsk	2.94	2.79	2.94	2.73	3.08	6	0.00014
VX770 + Forsk	4.56	4.56	4.69	4.38	4.73	6	0.00014
VX661 + Forsk	4.08	3.38	4.57	3.09	4.6	7	0.00015
VX445 + Forsk	4.54	4.36	4.74	4.08	5.06	4	0.00223
VX445 + VX661 + VX770 + Forsk	4.05	3.61	4.59	2.95	4.92	8	0.00013
PPQ102	0.91	0.84	0.94	0.67	1.07	10	0.00017
PPQ102 + Forsk	0.56	0.55	0.57	0.53	0.57	7	0.00043

Table S4. Transepithelial fluid flux (J) in CAPAN-1 epithelial models under basal, inflammatory, and pharmacological modulation conditions. Data are reported as median, interquartile range (IQR25–IQR75), minimum (MIN), and maximum (MAX) values. CAPAN-1 cells were treated with vehicle (DMSO), lipopolysaccharide (LPS), forskolin (Forsk), CFTR modulators (VX770, VX661, VX445) in combination with forskolin, or the CFTR inhibitor PPQ102, in the presence or absence of Forsk and LPS. Sample size (n) and p values (Kruskal-Wallis test followed by Dunn's multiple comparison test) are indicated. Comparisons were performed against the corresponding DMSO condition within each experimental group. Statistical significance was set at $p < 0.05$

Transepithelial fluid flux (J) in CAPAN-1 epithelial models (µL/(h·cm²))							
**TREATMENT**	**Median**	**IQR25**	**IQR75**	**MIN**	**MAX**	**n**	**p**
DMSO	2.07	2.05	2.07	2.02	2.08	10	-
DMSO+LPS	1.35	1.148	1.35	0.72	1.66	12	0.00077
Forsk	2.45	2.34	2.45	2.33	2.56	5	0.00053
Forsk+LPS	1.68	1.55	1.68	1.51	2.26	6	0.00027
VX770+Forsk	2.59	2.57	2.59	2.56	2.81	7	0.00044
VX770+Forsk+LPS	1.46	1.45	1.47	1.40	1.47	4	0.00196
VX661+Forsk	2.58	2.56	2.58	2.5	2.66	5	0.00054
VX661+Forsk+LPS	1.39	1.30	1.39	1.18	1.53	12	0.00012
VX445+Forsk	2.67	2.63	2.67	2.52	2.72	6	0.00015
VX445+Forsk+LPS	1.39	1.28	1.39	1.24	1.69	12	0.00012
VX770+VX661 +VX445+Forsk	2.65	2.54	2.65	2.44	3.08	5	0.00054
VX770+VX661 +VX445+Forsk+LPS	1.41	1.26	1.41	1.08	1.58	10	0.00038
PPQ102	1.90	1.87	1.9	1.85	2.01	5	0.00054
PPQ102+LPS	1.16	1.15	1.18	1.07	2.05	9	0.00017
PPQ102+Forsk	1.70	1.57	1.7	1.44	1.85	5	0.00054
PPQ102+Forsk+LPS	0.80	0.70	0.81	0.69	0.82	5	0.00048

Table S5. Apical surface fluid pH in HBE epithelial models under basal and pharmacological modulation conditions. Data are reported as median, interquartile range (IQR25-IQR75), minimum (MIN), and maximum (MAX) values. HBE cells were treated with vehicle (DMSO), forskolin (Forsk), CFTR modulators (VX770, VX661, VX445) in combination with forskolin, or the CFTR inhibitor PPQ102. Sample size (n) and p values (Kruskal-Wallis test followed by Dunn's multiple comparison test) are indicated. Comparisons were performed against the corresponding DMSO condition within each experimental group. Statistical significance was set at $p < 0.05$.

**pH of the apical surface fluid of HBE epithelial models**							
**TREATMENT**	**Median**	**IQR25**	**IQR75**	**MIN**	**MAX**	**n**	**p**
DMSO	7.24	7.15	7.41	6.95	8.00	9	-
Forsk	7.61	7.49	7.62	7.44	7.64	6	0.00275
VX770 + Forsk	7.43	7.34	7.58	7.29	7.68	6	0.02145
VX661 + Forsk	7.45	7.42	7.49	7.42	7.51	4	0.02123
VX445 + Forsk	7.42	7.37	7.45	7.34	7.52	6	0.03266
VX445 + VX661 + VX770 + Forsk	7.48	7.46	7.50	7.41	7.54	4	0.02699
PPQ102	7.07	6.92	7.12	6.80	7.14	7	0.04334
PPQ102 + Forsk	6.94	6.87	6.98	6.74	7.13	7	0.00392

Table S6. Apical surface fluid pH in CAPAN-1 epithelial models under basal, inflammatory, and pharmacological modulation conditions. Data are reported as median, interquartile range (IQR25-IQR75), minimum (MIN), and maximum (MAX) values. CAPAN-1 cells were treated with vehicle (DMSO), lipopolysaccharide (LPS), forskolin (Forsk), CFTR modulators (VX770, VX661, VX445) in combination with forskolin, or the CFTR inhibitor PPQ102, in the presence or absence of LPS. Sample size (n) and p values (Kruskal-Wallis test followed by Dunn's multiple comparison test) are indicated. Comparisons were performed using DMSO conditions as reference. Statistical significance was set at $p < 0.05$.

pH of the apical surface fluid of CAPAN-1 epithelial models							
**TREATMENT**	**Median**	**IQR25**	**IQR75**	**MIN**	**MAX**	**n**	**p**
DMSO	7.30	7.24	7.30	7.16	7.36	12	-
DMSO+LPS	7.17	7.12	7.17	7.06	7.30	5	0.00223
Forsk	7.52	7.50	7.52	7.47	7.55	5	0.00057
Forsk+LPS	7.26	7.23	7.26	7.21	7.38	5	0.53301
VX770+FORSK	7.62	7.61	7.62	7.59	7.65	5	0.00057
VX770+Forsk+LPS	7.38	7.33	7.38	7.32	7.49	5	0.00932
VX661+Forsk	7.43	7.43	7.43	7.43	7.52	5	0.00054
VX661+Forsk+LPS	7.24	7.11	7.24	6.99	7.59	5	0.69926
VX445+Forsk	7.45	7.40	7.45	7.40	7.54	5	0.00056
VX445+Forsk+LPS	7.21	7.15	7.21	7.12	7.27	4	0.12321
VX770+VX661 +VX445+Forsk	7.47	7.43	7.47	7.38	7.60	4	0.00221
VX770+VX661 +VX445+Forsk+LPS	7.22	7.19	7.22	7.15	7.38	5	0.35931
PPQ102	7.28	7.23	7.28	7.23	7.33	5	0.62532
PPQ102+LPS	7.12	7.11	7.12	6.81	7.18	5	0.00192
PPQ102+Forsk	7.00	6.80	7.00	6.44	7.18	12	0.00015
PPQ102+Forsk+LPS	6.94	6.72	6.94	6.51	7.31	6	0.00808

Table S7. Microviscosity of the apical surface fluid of HBE epithelial models. Data are reported as median, interquartile range (IQR25-IQR75), minimum (MIN), and maximum (MAX) values. HBE cells were treated with vehicle (DMSO), forskolin (Forsk), CFTR modulators (VX770, VX661, VX445, alone or in combination) in the presence of forskolin, or the CFTR inhibitor PPQ102, alone or in combination with forskolin. Sample size (n) and p values (Kruskal-Wallis test followed by Dunn's multiple comparison test) are indicated. Comparisons were performed using DMSO conditions as reference. Statistical significance was set at $p < 0.05$.

**Microviscosity of the apical surface fluid of HBE epithelial models (cPoise)**							
**TREATMENT**	**Median**	**IQR25**	**IQR75**	**MIN**	**MAX**	**n**	**p**
DMSO	1.52	1.4725	1.55	1.42	1.55	4	-
Forsk	1.31	1.27	1.3325	1.21	1.34	4	0.02197
VX770 + Forsk	1.25	1.185	1.255	1.12	1.26	3	0.04667
VX661 + Forsk	1.225	1.185	1.2725	1.14	1.34	4	0.02197
VX445 + Forsk	1.26	1.2425	1.2725	1.22	1.28	4	0.02048
VX445 + VX661 + VX770 + Forsk	1.24	1.23	1.26	1.2	1.32	4	0.00418
PPQ102	1.865	1.825	1.965	1.78	1.98	8	0.00288
PPQ102 + Forsk	2.56	2.3925	2.6975	2.07	2.91	10	0.02197

Table S8. Microviscosity of the apical surface fluid of CAPAN-1 epithelial models. Data are reported as median, interquartile range (IQR25-IQR75), minimum (MIN), and maximum (MAX) values. CAPAN-1 cells were treated with vehicle (DMSO), lipopolysaccharide (LPS), forskolin (Forsk), CFTR modulators (VX770, VX661, VX445) in combination with forskolin, or the CFTR inhibitor PPQ102, in the presence or absence of LPS. Sample size (n) and p values (Kruskal-Wallis test followed by Dunn's multiple comparison test) are indicated. Comparisons were performed using DMSO conditions as reference. Statistical significance was set at $p < 0.05$

Microviscosity of the apical surface fluid of CAPAN-1 epithelial models (cPoise)							
**TREATMENT**	**Median**	**IQR25**	**IQR75**	**MIN**	**MAX**	**n**	**p**
DMSO	1.98	1.98	1.99	1.98	1.99	5	-
DMSO+LPS	2.03	2.02	2.03	2	2.07	5	0.00432
Forsk	1.39	1.3675	1.3925	1.3	1.4	5	0.00432
Forsk+LPS	1.51	1.505	1.515	1.5	1.52	5	0.00406
VX770+FORSK	1.13	1.125	1.135	1.12	1.14	5	0.00406
VX770+Forsk+LPS	1.25	1.235	1.25	1.22	1.25	5	0.00432
VX661+Forsk	1.38	1.35	1.39	1.34	1.4	5	0.00458
VX661+Forsk+LPS	1.4785	1.425	1.48	1.365	1.57	5	0.00458
VX445+Forsk	1.39	1.3675	1.3925	1.3	1.4	5	0.00432
VX445+Forsk+LPS	1.51	1.505	1.515	1.5	1.52	5	0.00406
VX770+VX661 +VX445+Forsk	1.39	1.3675	1.3925	1.3	1.4	5	0.00432
VX770+VX661 +VX445+Forsk+LPS	1.4575	1.42	1.57	1.32	1.6	5	0.00458
PPQ102	1.95	1.91	1.96	1.9	1.98	5	0.01884
PPQ102+LPS	2.1	2.07	2.13	2	2.48	5	0.00458
PPQ102+Forsk	2.025	2.005	2.04	1.96	2.07	4	0.22852
PPQ102+Forsk+LPS	2.17	2.155	2.2775	2.11	2.6	4	0.00892

Table S9. Evaluation of WT CFTR protein expression in whole-cell lysates from HBE and CAPAN-1 epithelial layers. Western blot bands corresponding to total CFTR protein (sum of bands B + C) and maturation rate (ratio C/(C+B)) were quantified by densitometric analysis using ImageJ software. Band intensities were normalized to the housekeeping protein actin and expressed relative to DMSO-treated, LPS-unstimulated samples (set as 1). Data are reported as median, interquartile range (IQR25-IQR75), minimum (MIN), and maximum (MAX) values. Sample size (n) and p values (Kruskal-Wallis test followed by Dunn's multiple comparison test) are indicated. Comparisons were performed using DMSO-treated, LPS unstimulated condition as reference. Statistical significance was set at $p < 0.05$.

**WT-CFTR Total protein (C + B bands) in HBE cells**							
**TREATMENT**	**Median**	**IQR25**	**IQR75**	**MIN**	**MAX**	**n**	**p**
DMSO	1	0.99	1	0.99	1.03	4	-
Forsk	0.96	0.91	0.96	0.82	1.07	4	0.25824
VX770+VX661 +VX445+Forsk	0.93	0.85	0.93	0.79	1.05	4	0.30195
PPQ102+Forsk	0.90	0.88	0.9	0.84	1.03	4	0.25613

**WT-CFTR maturation rate (C/(C + B) bands) in HBE cells**							
**TREATMENT**	**Median**	**IQR25**	**IQR75**	**MIN**	**MAX**	**n**	**p**
DMSO	0.99	0.99	0.99	0.98	1.02	4	-
Forsk	1.01	0.96	1.01	0.89	1.09	4	0.74158
VX770+VX661 +VX445+Forsk	0.95	0.91	0.95	0.87	0.99	4	0.1109
PPQ102+Forsk	1.01	0.97	1.01	0.89	1.10	4	0.62921

**WT-CFTR Total protein (C + B bands) in CAPAN-1 cells**							
**TREATMENT**	**Median**	**IQR25**	**IQR75**	**MIN**	**MAX**	**n**	**p**
DMSO	1.05	1.02	1.05	1.00	1.20	5	-
DMSO+LPS	1.12	0.88	1.12	0.86	1.19	5	0.82384
Forsk	1.00	0.99	1.00	0.95	1.05	5	0.11428
Forsk+LPS	1.12	0.99	1.12	0.9	1.32	5	0.82384
VX770+VX661 +VX445+Forsk	1.00	0.99	1.00	0.95	1.05	5	0.035445
VX770+VX661 +VX445+Forsk+LPS	1.12	0.99	1.12	0.90	1.32	5	0.01663
PPQ102+Forsk	1.17	1.13	1.17	1.04	1.25	5	0.82384

PPQ102+Forsk+LPS	1.13	1.04	1.13	0.9	1.16	5	0.11428

WT-CFTR maturation rate (C/(C + B) bands) in CAPAN-1 cells							
**TREATMENT**	**Median**	**IQR25**	**IQR75**	**MIN**	**MAX**	**n**	**p**
DMSO	1.00	0.98	1.00	0.90	1.21	5	-
DMSO+LPS	0.99	0.90	0.99	0.84	1.33	5	0.72897
Forsk	1.21	0.93	1.21	0.72	1.67	4	0.82384
Forsk+LPS	1.14	1.05	1.14	0.94	1.37	4	0.82384
VX770+VX661 +VX445+Forsk	1.02	1.00	1.02	0.98	1.10	5	0.47132
VX770+VX661 +VX445+Forsk+LPS	0.83	0.81	0.83	0.79	1.00	5	0.82317
PPQ102+Forsk	0.83	0.81	0.83	0.79	1.01	5	0.11644
PPQ102+Forsk+LPS	0.83	0.81	0.83	0.79	1.01	5	0.14073

Table S10. Evaluation of IL-6, IL-8, and TNF-α expression in the basolateral medium of CAPAN-1 epithelial layers. Epithelial preparations were treated for 24 h with vehicle (DMSO), forskolin (Forsk), VX770+VX661+VX445 in combination with forskolin, or the CFTR inhibitor PPQ102 in combination with forskolin, in the absence or presence of LPS stimulation. Cytokine levels in the basolateral medium were assessed by Western blot analysis. To ensure comparability across lanes, total protein loading was verified by Coomassie blue staining. Band intensities were quantified by densitometric analysis using ImageJ software and normalized to total protein levels. Values are expressed relative to DMSO-treated samples without LPS, set as 1. Data are reported as median, interquartile range (IQR25–IQR75), minimum (MIN), and maximum (MAX) values. Sample size (n) and p values are indicated. Comparisons were performed using the DMSO-treated, LPS-unstimulated condition as reference. Statistical significance was set at $p < 0.05$.

IL-6							
**TREATMENT**	**Median**	**IQR25**	**IQR75**	**MIN**	**MAX**	**n**	**p**
DMSO	1.00	0.98	1.00	0.95	1.02	5	-
DMSO+LPS	1.35	1.31	1.35	1.30	1.53	5	0.00570
Forsk	1.00	0.93	1.00	0.88	1.29	5	0.82249
Forsk+LPS	1.33	1.29	1.33	1.27	1.37	5	0.00570
VX770+VX661 +VX445+Forsk	0.97	0.96	0.97	0.94	1.12	5	0.82351
VX770+VX661 +VX445+Forsk+LPS	1.41	1.37	1.41	1.33	1.65	5	0.00570
PPQ102+Forsk	0.99	0.96	0.99	0.89	1.17	5	0.82351
PPQ102+Forsk+LPS	1.44	1.30	1.44	1.25	1.59	5	0.00570
**IL-8**							
**TREATMENT**	**Median**	**IQR25**	**IQR75**	**MIN**	**MAX**	**n**	**p**
DMSO	1.00	0.98	1.00	0.98	1.07	5	-
DMSO+LPS	1.49	1.40	1.49	1.33	1.60	5	0.00570
Forsk	1.02	0.95	1.02	0.89	1.10	5	0.82351
Forsk+LPS	1.55	1.45	1.55	1.43	1.72	5	0.00570
VX770+VX661 +VX445+Forsk	1.01	1.00	1.01	0.99	1.12	5	0.47036
VX770+VX661 +VX445+Forsk+LPS	1.55	1.45	1.55	1.32	1.69	5	0.00570
PPQ102+Forsk	0.93	0.89	0.93	0.87	1.23	5	0.47036
PPQ102+Forsk+LPS	1.66	1.57	1.66	1.56	1.82	5	0.00570

TNF-α							
**TREATMENT**	**Median**	**IQR25**	**IQR75**	**MIN**	**MAX**	**n**	**p**
DMSO	1.00	1.00	1.00	0.96	1.20	5	-
DMSO+LPS	1.52	1.45	1.52	1.39	1.63	5	0.00570
Forsk	0.97	0.89	0.97	0.60	1.20	5	0.46940
Forsk+LPS	1.55	1.42	1.55	1.42	1.68	5	0.00541
VX770+VX661 +VX445+Forsk	1.07	0.96	1.07	0.75	1.20	5	0.82249
VX770+VX661 +VX445+Forsk+LPS	1.59	1.45	1.59	1.37	1.75	5	0.00570
PPQ102+Forsk	0.89	0.81	0.89	0.61	1.30	5	0.33307
PPQ102+Forsk+LPS	1.54	1.47	1.54	1.47	1.60	5	0.00541

Figure S1. Chemical structures of the CFTR modulators and inhibitor used in this study. Chemical structures of (A) ivacaftor (VX770), (B) tezacaftor (VX661), (C) elexacaftor (VX445), and (D) the CFTR inhibitor PPQ102. VX770 acts as a CFTR potentiator, whereas VX661 and VX445 are CFTR correctors. PPQ102 was used as a pharmacological inhibitor of CFTR-dependent transport. The 2D chemical structures of VX770, VX661, VX445, and PPQ102 are reported in Figure S2 and were obtained from PubChem (National Center for Biotechnology Information, National Library of Medicine).

A

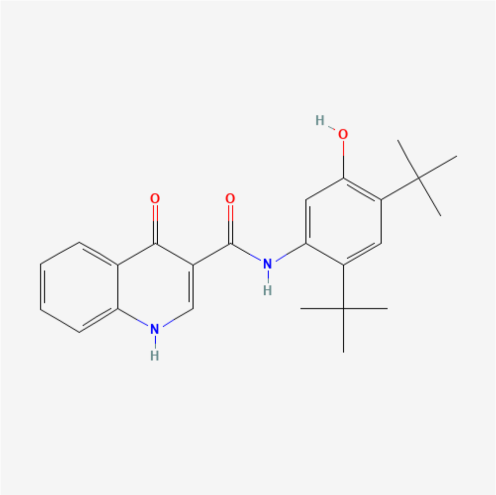

Ivacaftor (VX770)
N-(2,4-ditert-butyl-5-hydroxyphenyl)-4-oxo-1H-quinoline-3-carboxamide

B

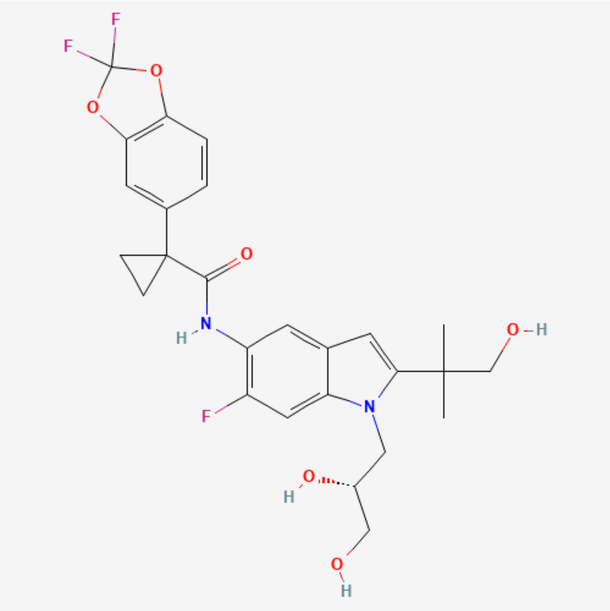

Tezacaftor (VX661)
1-(2,2-difluoro-1,3-benzodioxol-5-yl)-N-[1-[(2R)-2,3-dihydroxypropyl]-6-fluoro-2-(1-hydroxy-2-methylpropan-2-yl)indol-5-yl]cyclopropane-1-carboxamide

C

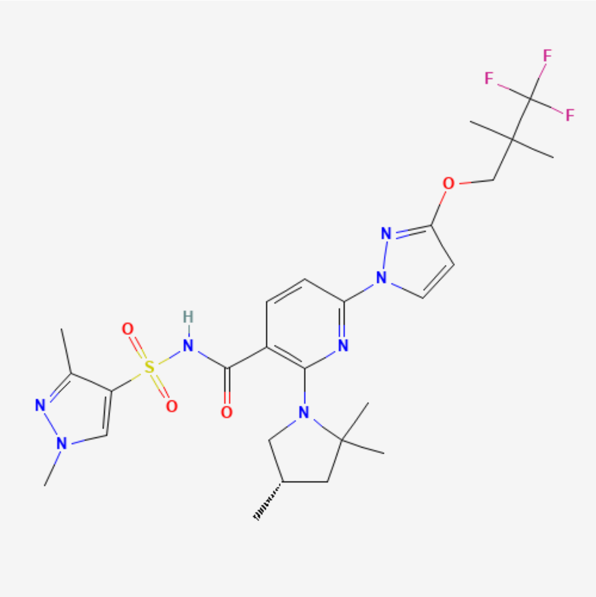

elexacaftor (VX445)
N-(1,3-dimethylpyrazol-4-yl)sulfonyl-6-[3-(3,3,3-trifluoro-2,2-dimethylpropoxy)pyrazol-1-yl]-2-[(4S)-2,2,4-trimethylpyrrolidin-1-yl]pyridine-3-carboxamide

D

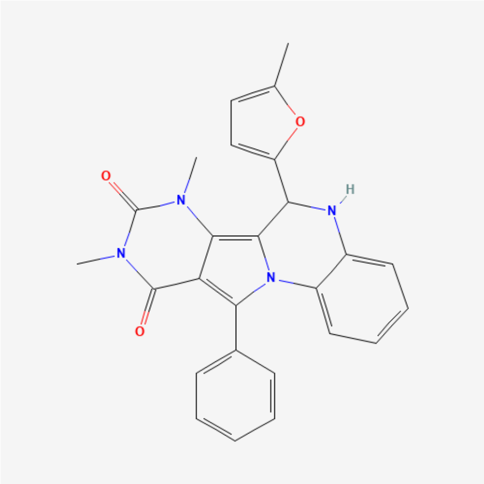

PPQ102
12,14-dimethyl-9-(5-methylfuran-2-yl)-17-phenyl-1,8,12,14 tetrazatetracyclo[8.7.0.02,7.011,16]heptadeca-2,4,6,10,16-pentaene-13,15-dione

Figure S2. Representative Coomassie blue-stained SDS-PAGE gel from concentrated basolateral medium of CAPAN-1 epithelial layers. Cells were treated with vehicle (DMSO), forskolin (Forsk), VX770+VX661+VX445 in combination with forskolin, or the CFTR inhibitor PPQ102 in combination with forskolin, in the absence (-) or presence (+) of LPS stimulation. Coomassie staining was performed to visualize total protein patterns and was used as a loading control to verify uniform protein loading across lanes. The densitometric signal was used for normalization of cytokine band intensities in the corresponding Western blot analyses. Molecular weight markers (kDa) are indicated on the left.

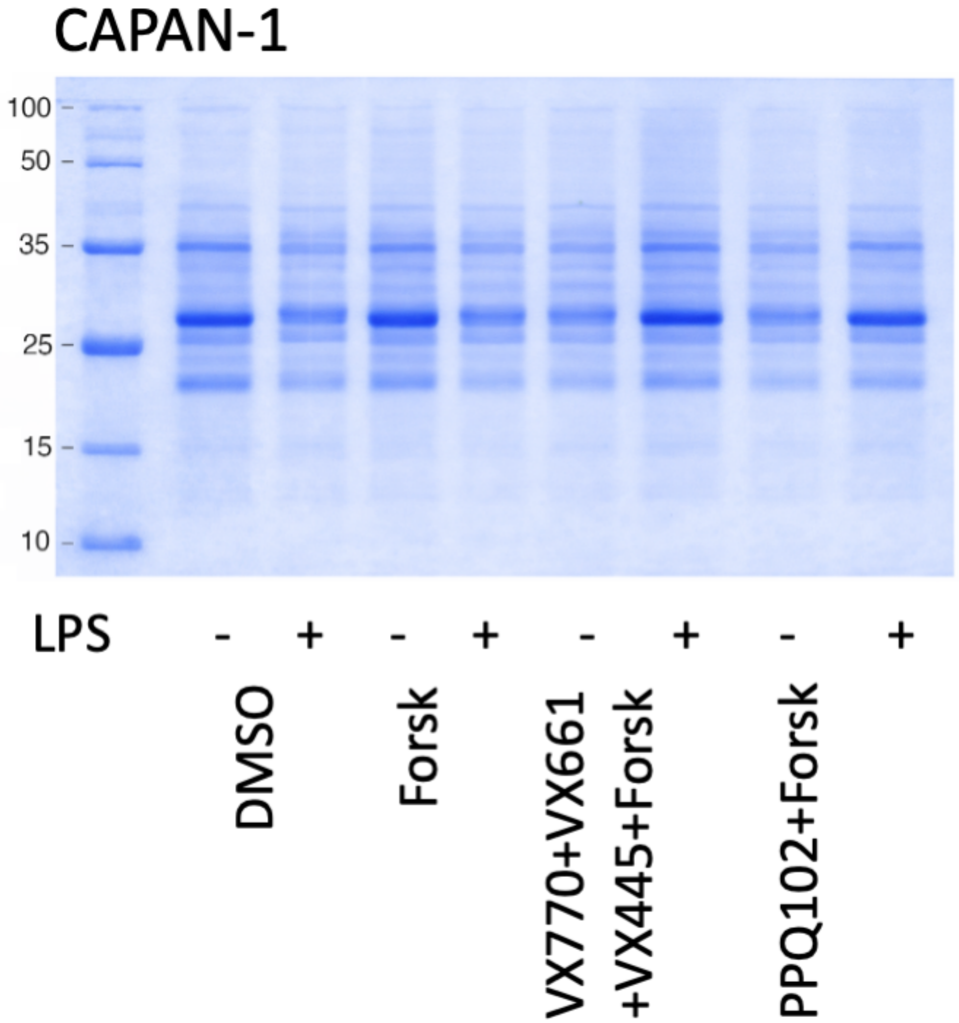

Supplement: Supplementary file 1 [file ijms-27-04868-s001.zip › ijms-4317658-supplementary.pdf]
